# The utility of herbarium collections for genetic monitoring

**DOI:** 10.1093/biosci/biag048

**Published:** 2026-05-06

**Authors:** Isaac Eckert, Lucas Eckert, Olivia Rahn, Cameron So, Simon Joly, Laura J Pollock

**Affiliations:** Department of Biology, Stewart Biology Building, 1205 Docteur Penfield, McGill University, Montréal, Québec H3A 1B1, Canada; Québec Centre for Biodiversity Science, Stewart Biology Building S3/18, 1205 Docteur Penfield, Montréal, Québec H3A 1B1, Canada; Department of Biology, Stewart Biology Building, 1205 Docteur Penfield, McGill University, Montréal, Québec H3A 1B1, Canada; Québec Centre for Biodiversity Science, Stewart Biology Building S3/18, 1205 Docteur Penfield, Montréal, Québec H3A 1B1, Canada; Department of Biology, Stewart Biology Building, 1205 Docteur Penfield, McGill University, Montréal, Québec H3A 1B1, Canada; Québec Centre for Biodiversity Science, Stewart Biology Building S3/18, 1205 Docteur Penfield, Montréal, Québec H3A 1B1, Canada; Department of Biology, Stewart Biology Building, 1205 Docteur Penfield, McGill University, Montréal, Québec H3A 1B1, Canada; Québec Centre for Biodiversity Science, Stewart Biology Building S3/18, 1205 Docteur Penfield, Montréal, Québec H3A 1B1, Canada; Québec Centre for Biodiversity Science, Stewart Biology Building S3/18, 1205 Docteur Penfield, Montréal, Québec H3A 1B1, Canada; Institut de recherche en biologie végétale and Département de Sciences biologiques, Université de Montréal, 4101 Sherbrooke East, Montréal, Québec H1X 2B2, Canada; Montreal Botanical Garden, 4101 Sherbrooke East, Montréal, Québec H1X 2B2, Canada; Department of Biology, Stewart Biology Building, 1205 Docteur Penfield, McGill University, Montréal, Québec H3A 1B1, Canada; Québec Centre for Biodiversity Science, Stewart Biology Building S3/18, 1205 Docteur Penfield, Montréal, Québec H3A 1B1, Canada

**Keywords:** herbarium collections, genetic diversity, genetic composition, essential biodiversity variables, genetic monitoring

## Abstract

Despite growing evidence of widespread genetic responses to anthropogenic activity, data shortfalls constrain genetic monitoring efforts and preclude the widespread use of genetic data to inform conservation. For flora, one option is to leverage the wealth of genetic material preserved in Earth’s vast herbarium collections, but the extent to which herbarium specimens can supply the population-level data required to monitor genetic change remains unclear. Using the Essential Biodiversity Variable (EBV) framework developed to monitor population-level genetic change, we show that digitized herbarium specimens could be used to quantify ∼162 K measures of genetic EBVs representing over 41 K species, 86% of regions on Earth, and spanning the past 250 years of global change. As such, we find that herbarium collections offer an invaluable source of historical genetic data, the mobilization of which could transform global efforts to monitor and conserve plant diversity.

Efforts to protect biodiversity are underpinned by our ability to detect and attribute change, which itself relies on the quantity and coverage of available biodiversity data (Gonzalez et al. 2023). Despite rapid increases in the amount of data being collected (König et al. 2019) and calls for additional monitoring to meet national and international conservation targets (Kühl et al. 2020), critical data shortfalls persist (Hortal et al. 2015), limiting our ability to describe biodiversity and detect change at large spatial, temporal, or taxonomic scales. This is especially true when it comes to our knowledge of *genetic composition* (Hoban et al. 2022), which comprises multiple aspects of within-species genetic variation that influence species’ capacities for adaptation (Lande and Shannon 1996, Sgrò et al. 2011). Given direct relationships to extinction risk (Kardos et al. 2021) and growing evidence of widespread genetic change (Shaw et al. 2025), monitoring change in genetic composition is increasingly viewed as critical to global biodiversity conservation (DeWoody et al. 2021, United Nations Convention on Biological Diversity 2022). To that end, four Essential Biodiversity Variables (EBVs: *Genetic Diversity, Genetic Differentiation, Inbreeding, and Effective Population Size*; Pereira et al. 2013) have been proposed to standardize monitoring efforts and inform conservation (table 1; see also Hoban et al. 2022). However, global shortfalls in the availability of population-level genetic data—which are required to robustly monitor change in genetic EBVs—prevent their widespread application (Hoban et al. 2021, 2022).

**Table 1.** Genetic EBVs, their definitions, sample size requirements *(individuals per population)*, and conservation use.

EBV	General definition	Example metrics	Min sample size per population	Conservation relevance
**Genetic diversity**	Diversity of alleles or heterozygotes in a population (e.g., richness or evenness)	π, **H_e_,H_o_**	**8**	Change indicates shifts in adaptive potential
**Genetic differentiation**	Differentiation between populations (e.g., number of lineages or degree of differentiation)	**F_ST_**	**8**	Change indicates shifts in connectivity, long term persistence, and the potential loss of adaptations
**Inbreeding**	Degree of relatedness among parents of individuals in a population	**F_IS_**	**30**	High inbreeding is associated with a decrease in fitness through expression of recessive deleterious alleles
**Effective population size**	Size of ideal population that loses genetic variation at the same rate as the sampled population	**N_e_**	**30**	Lower effective population size increases genetic drift, inbreeding, and future loss of genetic variation

For information on minimum sample size estimates, see box 1.

For flora, one way to address this data gap could be to mobilize the wealth of genetic data housed in Earth’s vast herbarium collections (Wandeler et al. 2007, Thiers 2024, Viveiros-Moniz et al. 2025). Over the past half millennia, humanity has amassed hundreds of millions of herbarium specimens, which are increasingly being recognized as a source of irreplaceable biodiversity data (Albani Rocchetti et al. 2021, Eckert et al. 2024, 2025a). Such specimens have been used to identify new species (Bebber et al. 2010), resolve shifting (Calinger 2015) or cryptic (Rosche et al. 2025) geographic distributions, sample functional traits (Heberling 2022) and biotic interactions (Meineke and Davies 2018), and track various aspects of biodiversity change (Meineke et al. 2018b, Johnson et al. 2011, 2018a) like phenology (Panchen et al. 2025). Now, thanks to the development of novel DNA extraction, sequencing, and bioinformatic workflows (Latorre et al. 2020, Marinček et al. 2022, Gouker et al. 2023), it is also possible to access the genetic data housed in these collections. Recognizing the unique potential of herbaria to supply historical genetic data, some studies are starting to take advantage of the broad temporal resolution of these specimens to monitor genetic change (Eckert et al. 2025b). Existing proposals even call for the broadscale application of herbarium collections to genetic monitoring, and proof of concepts demonstrate the feasibility of transforming specimens into genetic EBVs (Viveiros-Moniz et al. 2025). However, the distribution of specimens is uneven across time, space, and species (figure 1a–d), meaning the ability of herbarium collections to contribute the population-level data used in standardized genetic monitoring remains unclear (Eckert et al. 2025b).

**Figure 1.**
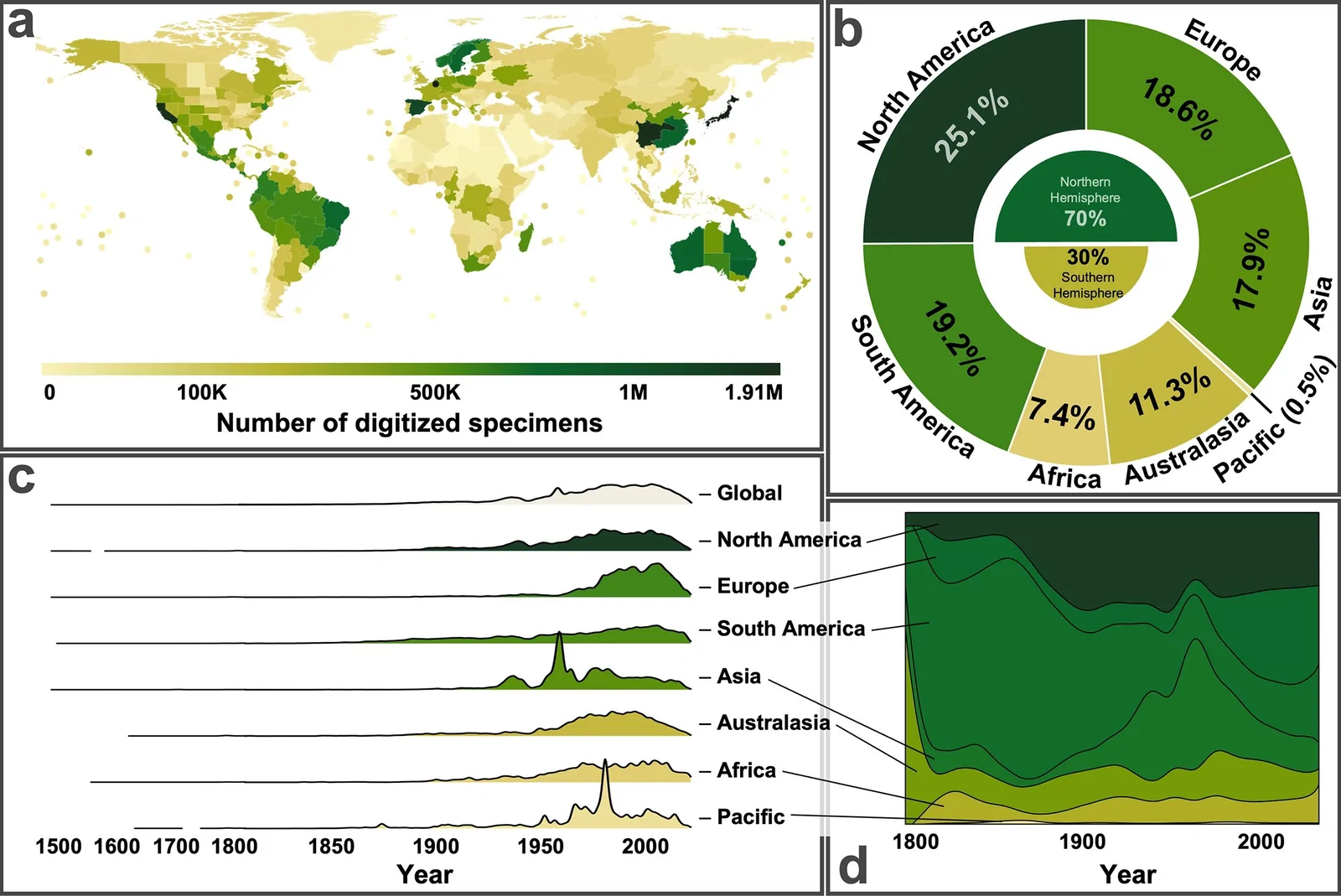
Distribution of Earth’s digitized herbarium specimens, which represent around 78% of extant plant species (Govaerts et al. 2021). The spatial distribution (a) of digitized specimens varies across botanical countries (the spatial delineations used by the World Geographic Scheme for Recording Plant Distributions) with clear biases toward California, Spain, eastern Brazil, Scandinavia, central China, and Australia. Across continents and hemispheres (b) North and South America boast the highest number of digitized specimens and around 70% of digital records originate from the Northern Hemisphere. Based on the relative density of collection through time (c), although some specimens date back to the 1500s, most were collected over the last century, with collection ramping up between 1950 and 2000 and steadily declining since; however, the relative frequency of collection across continents (d) is highly dynamic, likely due to variation in economic, political, and scientific development around the world.

In this paper, we build on past proposals (Eckert et al. 2025b) and proof of concepts (Viveiros-Moniz et al. 2025) by offering the first quantitative assessment of the ability of herbarium collections to supply population-level genetic data to monitor genetic EBVs for Earth’s vascular plants. To do so, we first formalize the requirements for herbarium specimens to contribute to standardized genetic monitoring using biologically conservative population limits informed by species functional traits. Next, we transform over 51 million digitized herbarium specimens housed in the Global Biodiversity Information Facility (GBIF) into spatially and temporally distinct populations of specimens that could be sequenced to supply the population-level genetic data required to quantify genetic EBVs (box 1). Then, to account for the fact that most herbarium specimens remain undigitized (Thiers 2024), we also estimate the potential benefits of specimen digitization when it comes to our ability to use herbarium collections in genetic monitoring (box 2). Finally, we discuss the potential implications of mobilizing this vast wealth of preserved genetic data, including how this data could benefit ecological and evolutionary research and conservation, the potential implications for herbaria, researchers, policy makers, and the public, as well as important considerations, limitations, and best practices for applying herbarium specimens to genetic monitoring. Together, our work provides the first estimate of the potential for Earth’s herbarium collections to contribute to our understanding of plant genetic composition and monitoring of genetic change globally.

Box 1. **Integrating collections and genetic monitoring: reconstructing populations from the herbarium record.**

To contribute to standardized genetic monitoring efforts, herbarium specimens need to be able to reconstruct plant populations—meaning that for a given species, there must be enough specimens from the same region and time period to provide the genetic data required to estimate population-level genetic EBVs (Hoban et al. 2022). To reconstruct populations, we first accessed all digitally available plant specimens (figure 2a) housed on the Global Biodiversity Information Facility (*n* = 51,230,881 accessed on 24/10/2024; GBIF.Org  2024).

**Figure 2.**
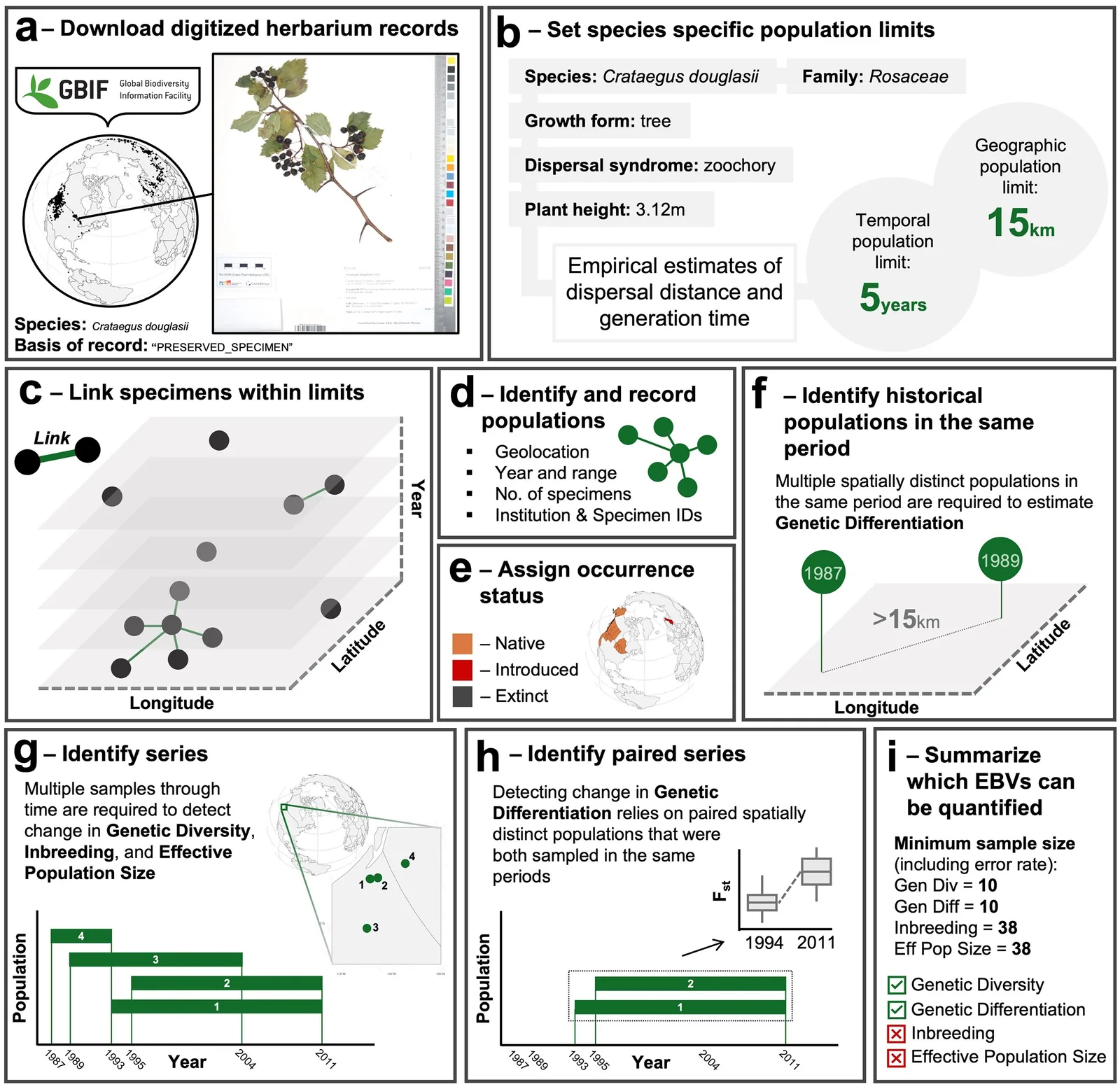
For a species of interest (a), we used population limits informed by commonly measured functional traits (b) to cluster digitized records into populations (c–d). Then, using these reconstructed populations, we then assigned occurrence status (e) and identified spatiotemporal replication (f–h) to assess which genetic EBVs could be quantified (i) if the specimens comprising these populations were sequenced.

Next, we needed to set geographic and temporal limits to dictate which digitized specimens clustered into the same population (figure 2b). In theory, robustly estimating most genetic EBVs relies on the assumption that sampled individuals represent panmictic populations, where individuals have an equal probability of mating (Charlesworth and Charlesworth 2010). In reality, ensuring panmixia is not straightforward (Landguth et al. 2012) and can be particularly challenging with herbarium specimens being fixed in time and space. That said, we can design approaches to increase the likelihood that genetic data obtained from herbaria is panmictic by reconstructing populations using biologically realistic and conservative spatial and temporal population limits informed by species’ functional traits (Paz-Vinas et al. 2021).

To inform geographic limits (max distance between specimens), we used dispersal estimates from Lososová and colleagues (2023) and filled missing data using genus, family, and growth form level averages, assigning geographic limits of 1, 5, and 15 km for poor, moderate, and good dispersers, respectively. For temporal limits (max years between specimens), we used modeled relationships between growth form and generation length (table S1; Compagnoni et al. 2021) to group species into annuals (Govaerts et al. 2021), “short,” and “long” generation plants, and assigned temporal limits of 1, 2, and 5 years, respectively. Although these limits are conservative (table S2) and potentially more restrictive than reality for some species (Savolainen et al. 2007, Millette et al. 2020), our approach helps maximize comparability across reconstructed populations.

Using these limits, we linked together specimens using a clustering approach to maximize the likelihood of forming biologically realistic populations (figure 2c and see figure S1; Paz-Vinas et al. 2021). To account for duplicate specimens (from the same individual plant), we identified and removed duplicated records with matching metadata (see Supplementary Section 1.6; Wilcox et al. 2025), before recording population information (figure 2d), and assigning occurrence status (figure 2e). For genetic differentiation, which requires at least 2 populations from the same period, we identified pairs of spatially distinct populations sampled from the same period (figure 2f).

These reconstructed populations have the potential to offer single *snapshots* of genetic EBVs in time and space. However, for many well sampled species and regions, enough specimens might exist at multiple points in time to allow us to assemble these *snapshots* into *series*. To do so, we identified and linked temporally distinct *snapshots* while ensuring geographic limits were not exceeded (figure 2g–h). Finally, we assessed which reconstructed populations contained enough specimens to enable robust estimates of genetic EBVs (figure 2i) based on conservative sample size requirements. For genetic diversity and differentiation, we required a minimum of 8 specimens and a minimum of 30 for inbreeding and effective population size (table 1; Nazareno et al. 2017, Gargiulo et al. 2024). We accounted for additional limitations (e.g., misidentification, inaccessibility, imperfect extraction/sequencing) by assuming that only 80% of digital records would produce exploitable genetic data (based on expert knowledge and Latorre et al. 2020, Sugita et al. 2020, Marinček et al. 2022, Gouker et al. 2023). Detailed methodology is available in the Supplementary (Section 1).

**Table 2.** Number and corresponding taxonomic coverage of potential snapshots and series across EBVs reconstructed from digitized herbarium records.

EBV	Measure (*n* = number of time points)	Number of measures possible	Number of species	Percentage of plant species
**Genetic Diversity**	Snapshot (*n* = 1)	162,636	41,642	11.7%
	Series (*n* = 2)	19,161	6282	1.8%
	Series (*n* ≥ 3)	2160	1571	0.4%
**Genetic Differentiation**	Snapshot (*n* = 1)	365,035	13,880	3.9%
	Series (*n* = 2)	3890	871	0.2%
	Series (*n* ≥ 3)	172	80	<0.1%
**Inbreeding and Effective Population Size**	Snapshot (*n* = 1)	8390	3999	1.1%
	Series (*n* = 2)	468	362	0.1%
	Series (*n* ≥ 3)	22	21	<0.1%

## Herbarium collections can be used to reconstruct hundreds of thousands of populations

**Figure 3.**
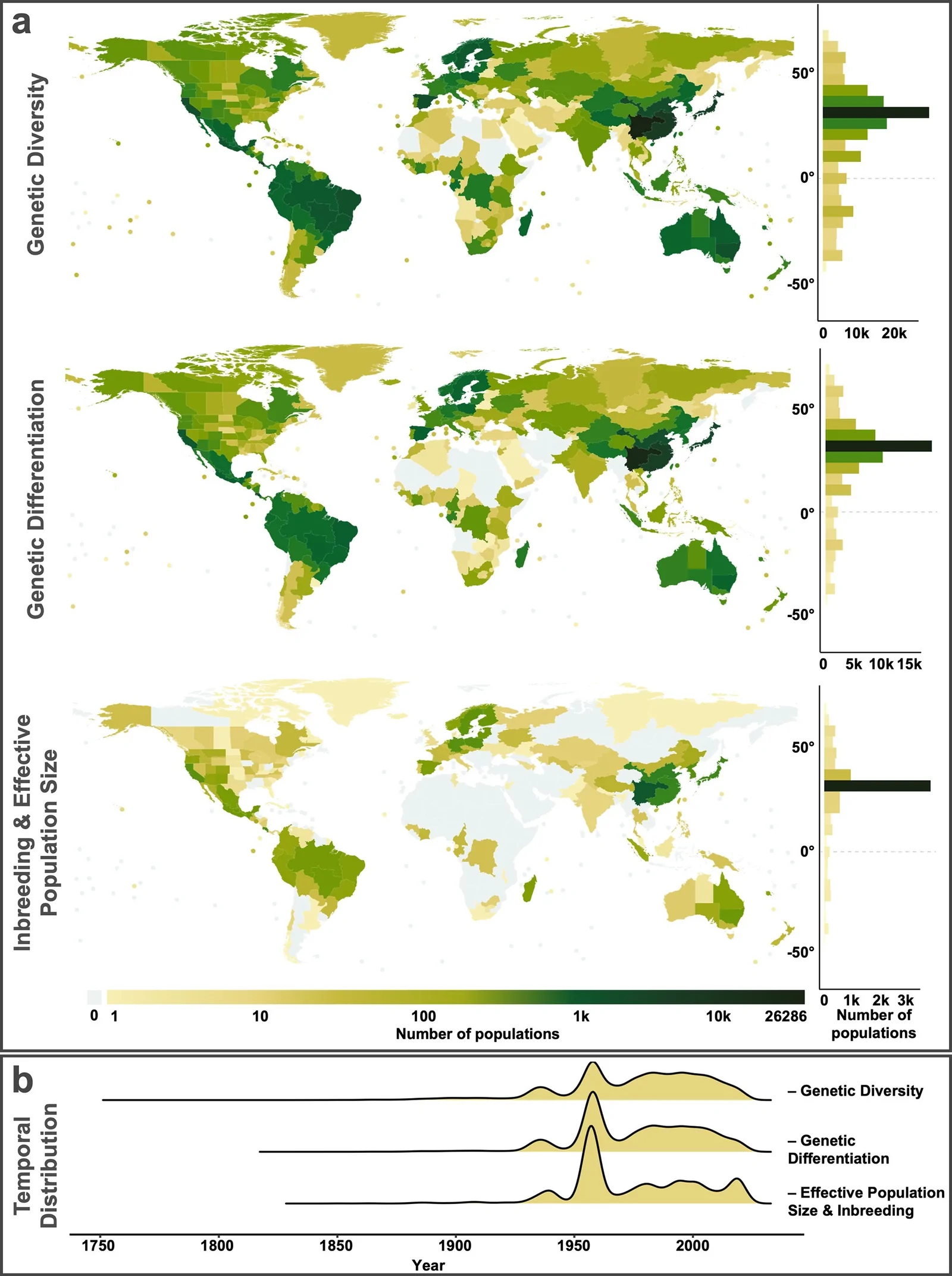
Spatial (a) and temporal (b) distributions of reconstructed populations that could be sequenced to quantify a snapshot of genetic EBVs. The spatial distribution largely reflected that of Earth’s herbarium specimens, with large numbers of reconstructed populations in Europe, North America, China, Brazil, Madagascar, Australia, and parts of Central and South America (notably the Andes and western Amazon) and central Africa. Although large regions of the world are poorly represented, only 51 out of Earth’s 369 WCVP botanical countries contained zero reconstructed populations. In general, spatial coverage for genetic diversity and differentiation was broader compared to inbreeding and effective population size, where regions across many continents and nearly all oceanic islands are very poorly represented. Temporally (b), most populations were collected post 1950. Interestingly, thanks to a short period of intense collection during China’s Great Leap Forward (Qin 1999), around 9% of all the populations we reconstructed were gathered between 1950 and 1970 in south-central China, representing over 3.7 thousand plant species.

Using our approach to reconstruct plant populations from the herbarium record (box 1, figure 2), we identified hundreds of thousands of populations with enough specimens to quantify *snapshots* and *series* of genetic EBVS (table 2). Compared to recent meta-analytical attempts to monitor genetic change across the tree of life (Shaw et al. 2025), the *series* we identified capture considerably longer timespans, represent far more species, and better represent historical time periods (see figure S2 and Supplementary Results for details). Surprisingly, the populations we were able to reconstruct have broad spatial and temporal coverage (figure 3), representing nearly all World Checklist of Vascular Plants (WCVP) botanical countries and dating back to as early as 1760.

**Figure 4.**
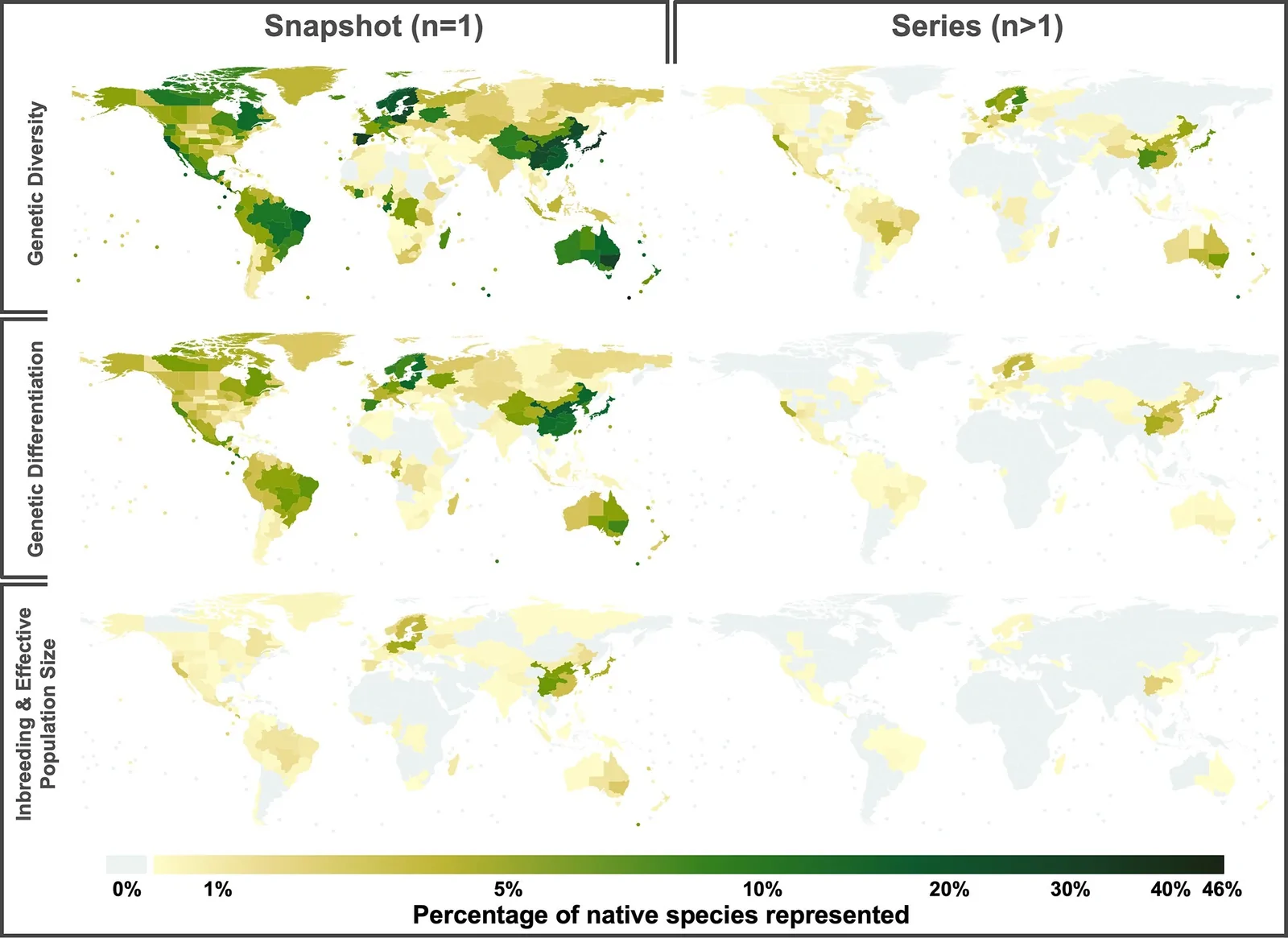
The taxonomic coverage (represented as the percentage of native species) of potential snapshots and series varies greatly across space and by EBV. For genetic diversity, the botanical countries with the best coverage include Macquarie Island in the South Pacific Ocean, Japan, Costa Rica, Spain, and New South Wales in Australia, where between 45% and 27% of native species are represented. Most botanical countries (77%) have less than 5% coverage of native species, which is unsurprising given that populations we identified only represent 11.7% of all vascular plant species. Taxonomic coverage was poorer for genetic differentiation and poorer still for effective population size and inbreeding. Moving from snapshots to series, only small fractions of plant communities are represented at regional scales regardless of EBV. Here, it is important to note that many species remain undescribed, especially in tropical megadiverse regions (Ondo et al. 2024), and so this representation may be overestimating coverage in those areas.

Our assessment suggests that mobilizing the genetic data stored in herbarium collections would substantially improve our knowledge of genetic composition and ability to monitor genetic change globally. For many regions, the reconstructed populations represent a considerable percentage of resident native species (figure 4). In these well sampled areas, resolving genetic trends across many species would gradually confer insights into community or ecosystem-wide patterns, benefiting conservation efforts to protect genetic composition and reduce extinction risk. That said, large data gaps would persist—digitized specimens only capture population-level genetic composition for 1%–12% of vascular plants depending on the EBV (table 2) and spatial coverage is highly variable, leaving many species and regions underrepresented.

**Figure 5.**
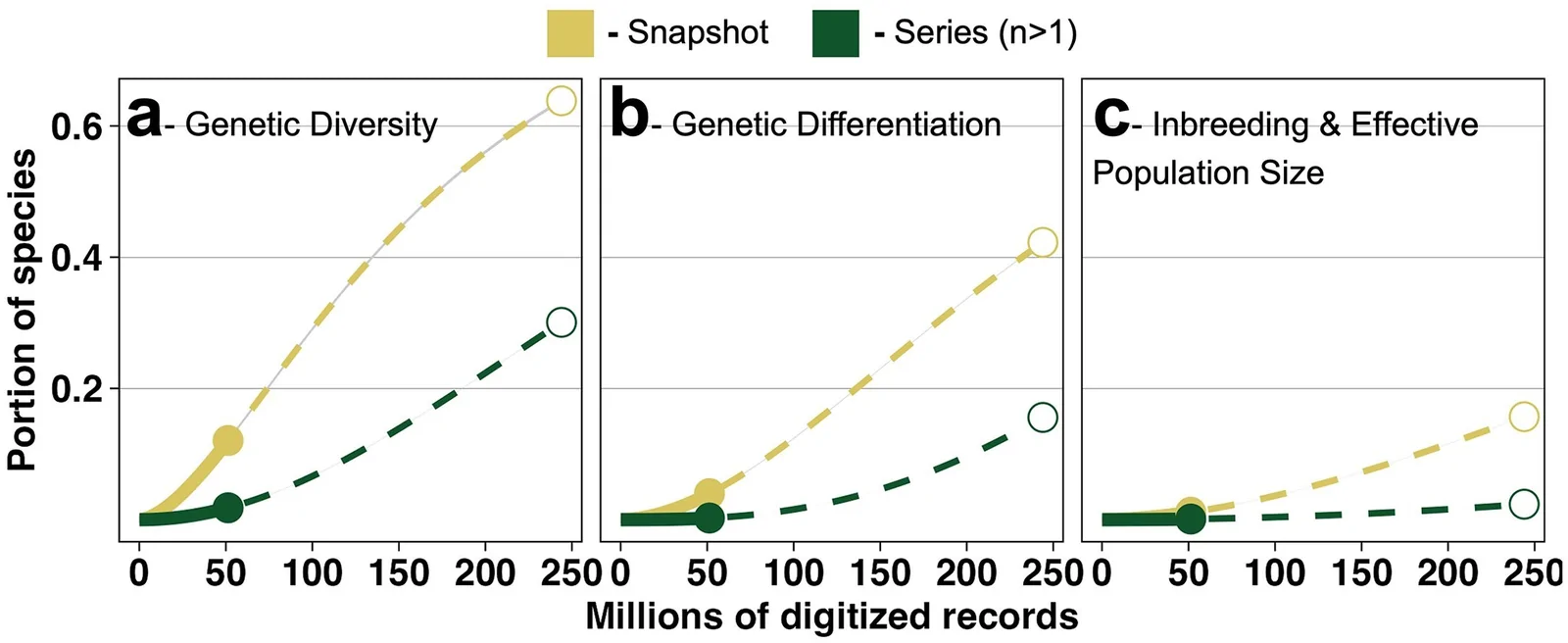
Based on the modeled rate at which these digitized specimens cluster into populations and accrue taxonomic coverage (visualized here using solid lines, tables S3–S8), we predict that the digitization of Earth’s remaining specimens would likely accrue large gains in the number of species for which we could reconstruct populations suitable for quantifying genetic EBVs. For genetic diversity (a), although we were able to reconstruct populations for 11.7% of all vascular plants from digitized collections, our results indicate that the mass digitization of remaining specimens could increase taxonomic coverage to 63.8%. For snapshots of genetic differentiation, effective population size and inbreeding, the potential increases are even greater (3.9% to 42.3% and 1.1% to 15.7%, respectively). Driven by poor existing taxonomic coverage and delayed initial rates of accumulation, the potential gains in the number of species represented in series (*n* = 2) are also substantial (1.8% to 30.1%, 0.2% to 15.6%, 0.1% to 2.4% for genetic diversity, differentiation, and inbreeding/effective population size, respectively; table S9).

When it comes to addressing these remaining data gaps, one option is to digitize more specimens. In the context of genetic monitoring, specimen digitization is a critical first step, allowing researchers to gauge the taxonomic, temporal, and spatial extent of available genetic material. Considering the digitally accessible records used in our analysis represent only a fraction (∼21%) of Earth’s total herbarium collections (Thiers 2024), we wanted to understand how the digitization of remaining specimens would benefit applications like genetic monitoring. Following past work (Eckert et al. 2024), we estimate that digitization has the potential to dramatically increase the taxonomic coverage of reconstructed populations (box 2), potentially even allowing us to obtain measures of genetic diversity for over half of extant plant species (figure 5). This shows that digitization is likely an effective approach to improving the extent and coverage of obtainable population-level genetic data, indicating that digitization initiatives have an important role to play when it comes to increasing the utility of herbarium collections to global change research (Eckert et al. 2024).

Box 2. **Estimating the potential benefits of digitization.**

To understand how digitization of additional specimens would affect our ability to apply herbarium collections to genetic monitoring initiatives, we built accumulation curves to predict how the mass digitization of Earth’s remaining specimens might increase the taxonomic coverage of reconstructed populations. Following Eckert and colleagues (2024), we randomly accumulated specimens while recording the rate at which taxonomic representation (species represented by populations or time series) increased. This process was repeated 1000 times to produce a distribution of accumulation curves that were used to fit zero-inflated beta regressions (Geissinger et al. 2022). Specifically, for each EBV, we built two distributions of accumulation curves to model both the accumulation of species represented by *snapshots* (*n* = 1) as well as species represented by *series* (*n* = 2). These models were then used to predict the increase in the portion of species represented given the digitization of Earth’s remaining specimens. Details are available in the Supplementary Material.

## The potential contribution of herbaria to genetic monitoring

Given direct links to extinction risk (Kardos et al. 2021), monitoring change in species genetic composition is invaluable when it comes to informing conservation initiatives and tracking progress. Accordingly, preserving genetic diversity of wild populations was included in the targets of the Kunming-Montreal Global Biodiversity Framework (Target 4; Convention on Biological Diversity 2022). Yet despite its inclusion, efforts to monitor genetic change remain inadequate (Hoban et al. 2023), even in data-rich regions of the world like Europe (Pearman et al. 2024). As such, recent synthesis (Csilléry et al. 2025) and meta-analytical (Shaw et al. 2025) efforts to compile trends in genetic data are severely limited in taxonomic, spatial, and temporal scope—leaving nearly all species and many regions unrepresented.

Our assessment suggests that herbarium collections could be instrumental in filling these data gaps for vascular plants. In total, we were able to reconstruct hundreds of thousands of populations with enough specimens in the herbarium record to permit estimation of genetic EBVs (table 2). The coverage of these reconstructed populations is surprisingly broad: they represent 11.7% of plant species, 86% of regions on Earth, and date back over 250 years. As such, the genetic data obtained from sequencing specimens from these populations could provide important baseline information to detect and attribute genetic change—offering researchers insight into genetic trends for tens of thousands of plant species. That said, population-level genetic data obtained from herbarium collections will come with limitations (box 3) and the extent to which this data can be seamlessly integrated alongside existing genetic monitoring frameworks and standards remains unclear.

Nonetheless, compared to current efforts, mobilizing the genetic data housed in Earth’s herbaria would dramatically increase the taxonomic and spatial coverage of available population-level genetic data for vascular plants (Csilléry et al. 2025). Importantly, digitization of remaining specimens has the potential to accrue additional taxonomic coverage—further increasing the utility of herbaria and their collections to genetic monitoring (figure 5). However, for many species and regions, biases present in herbarium collection will likely still preclude representation. For example, herbarium collections are often biased toward accessible areas, and to common or particularly interesting species (Daru et al. 2018, Lang et al. 2019), meaning that targeted sampling programs are likely necessary to ensure broadscale spatial and taxonomic coverage.

For time series, another option to rapidly generate data for unrepresented species is the contemporary resampling of populations identified in historical herbarium records. Based on recent herbarium and iNaturalist records, around 1/3 of the populations we identified have been observed in the past decade, meaning their persistence is likely (see supplementary for details). Future collection in these populations could add a contemporary time point that, combined with historical specimens, would allow us to monitor genetic change for an additional 1.5–12 K species depending on EBV (table S10). Taken together, we posit that herbaria and their collections can make substantial contributions to genetic monitoring efforts, filling genetic data gaps for tens of thousands of species, and improving our ability to detect and attribute past, contemporary, and future genetic change in Earth’s plants. That said, effectively transforming herbarium specimens into the genetic EBVs warrants specific considerations and is prone to a variety of limitations, many of which we discuss in box 3.

## Beyond genetic monitoring: novel insights from pressed and preserved genetic data

Although herbaria have historically played a foundational role in inventorying plant diversity, resolving phylogenies, and describing new species (Lane 1996), our findings suggest that the value and applications of these collections extend far beyond their original purpose (Heberling and Isaac 2017). As such, while we focused on the potential for herbaria to contribute to global genetic monitoring efforts, the utility of the genetic data housed in herbaria is much broader. Increasingly, studies are starting to leverage these genetic data to estimate historical baselines and test pressing ecological and evolutionary hypotheses regarding the drivers, patterns, and processes of genetic change in the Anthropocene (Eckert et al. 2025b).

For example, to explore the genetic mechanisms structuring biological invasion, Martin and colleagues (2014) sequenced specimens of *Ambrosia artemisiifolia* dating back over 140 years to resolve the species genetic response to land use change. Their analysis revealed that rapid expansion of agricultural land in North America likely led to the spread of *Ambrosia artemisiifolia* genotypes well adapted to human-disturbed landscapes, reducing genetic differentiation over time. The authors speculate that these genotypes are likely the ones responsible for founding some of the most aggressive populations of *A. artemisiifolia* in its introduced range across Europe—suggesting that invasiveness can evolve in a species native range through selection in response to anthropogenic activity. Expanding on this work, Bieker and colleagues (2022) sequenced over 300 herbarium specimens of *Ambrosia artemisiifolia* dating back over 190 years, which, at the time, constituted the largest use of conspecific genomic data from herbarium specimens for any species. Pairing these historical genomes with ones obtained from contemporary samples, the authors showed that invasiveness in *Ambrosia artemisiifolia* is probably driven by multiple factors, including admixture of source populations, the ability for invading individuals to hybridize with resident related species, and selection on defense genes because of escape from specific microbial enemies in the species introduced range.

In addition to revealing important mechanisms governing invasiveness, the work by Martin and colleagues (2014) and Bieker and colleagues (2022) also demonstrate the potential of herbarium collections to provide the broadscale genetic data required to resolve past biological invasions. Based on our results, we speculate that studies like those focusing on *Ambrosia artemisiifolia* are likely possible for thousands of other species around the world—we reconstructed over 6 K populations located in the introduced ranges of over 1.7 K species. In addition to providing estimates of genetic EBVs for monitoring initiatives, sequencing the specimens comprising these populations could also improve our understanding of the genetic component of biological invasion, including invasiveness, bottleneck effects, genetic drift, and rapid adaptation to novel environments (Bock et al. 2015).

Alongside biological invasions, recent work has also leveraged the historical genomes preserved in herbaria to understand the genetic causes and consequences of extinction. For example, Rosche and colleagues (2022) sequenced 81 specimens and over 300 contemporary samples of *Biscutella laevigata* to track changes in genetic diversity, differentiation, and inbreeding preceding and following extinction events. By pairing historical specimens with contemporary samples, they were able to show that while past local extinctions led to the irreversible loss of distinct genetic clusters, these events did not alter species-wide genetic diversity. Notably, prior to one population’s extinction, Rosche and colleagues (2022) managed to detect a significant decrease in observed heterozygosity (a metric of genetic diversity) from 9 specimens collected from 1862 to 1965. For persisting populations, no significant decline in heterozygosity was found, suggesting that monitoring declines in EBVs like genetic diversity could be used to forecast impending extinctions and prescribe conservation interventions to prevent them. Interestingly, we were able to reconstruct 66 historical populations across 34 species located in regions where species have since undergone extirpation. Sequencing these populations identified using our approach would allow researchers to build on the work of Rosche and colleagues (2022), by exploring the genetic signature of local extinction across more taxa and using more representative, population level (gene-pool) metrics of EBVs.

Past work has also focused on resolving patterns of genetic change in threatened species, which can provide critical information on the factors influencing extinction risk, dictate which species are considered endangered, and lead to direct conservation action to prevent biodiversity loss (Lande 1995, Kardos et al. 2021). For example, to understand the threats facing *Anacamptis palustris*, an endangered marsh orchid, Cozzolino and colleagues (2007) used historical specimens dating back over 150 years to piece together genetic changes in response to habitat loss and fragmentation. Interestingly, they found that many historical alleles had gone extinct, and many other alleles that used to be widespread are now only present in select populations, ultimately indicating that human induced habitat changes have caused declines in genetic diversity and significant increases in genetic differentiation over time, increasing the extinction risk facing *Anacamptis palustris*. Similarly, Nygaard and colleagues (2022) used historical specimens to understand why *Dracocephalum ruyschiana* experiences higher extinction risk in Norway compared to elsewhere in its range, surprisingly finding little evidence of genetic change through time or regional bottleneck effects, but showing that populations in Norway are genetically divergent, rendering their continued protection relevant. Taking advantage of *ex situ* conservation programs, Hansen and colleagues (2023) combined herbarium specimens with samples from living collections to resolve the genetic structure of *Gardenia remyi*, a critically endangered tree endemic to Hawaii. With fewer than 50 individuals left in the wild, gauging remaining genetic diversity and population structure is critical to ensuring the conservation of this species. The analysis by Hansen and colleagues (2023) detected clear divisions between genetic populations across islands as well as multiple subpopulations, providing important information for future conservation planning and breeding efforts to protect interspecific genetic diversity while minimizing inbreeding.

When it comes to at-risk flora, our assessment shows that herbarium collections could be potentially used to estimate genetic EBVs for 2447 species listed as threatened and 4583 species that are predicted (as per Bachman et al. 2024) to be threatened but have yet to be assessed. For these species, the genetic data stored in herbarium collections would not only contribute to global monitoring efforts but could also offer critical information on the capacity of these species to weather ongoing global change. This could ultimately help inform species-at-risk assessments (McLaughlin et al. 2025) and conservation strategies to mitigate extinction risk and prevent the loss of these imperilled taxa (Caballero et al. 2010).

In well studied species, researchers are starting to integrate the vast spatiotemporal distribution of available herbarium specimens alongside advanced understanding of genetic architectures, to enable detailed detection and attribution of genetic change. For example, to complement existing genetic data, Lopez and colleagues (2025) sequenced an additional 130 specimens of *Arabidopsis thaliana* to document the previously uncharacterized genetic diversity of populations across Africa. Together, their more comprehensive dataset revealed parallel patterns of genetic stasis and sporadic change in geographically disparate populations and helped resolve a more complete gradient of genetic diversity across *Arabidopsis thaliana*’s global range. Also working with *Arabidopsis thaliana*, Lang and colleagues (2024) combined genetic data from herbarium specimens with data on stomatal density, a functional trait that is expected to decrease in response to anthropogenic climate change. This integrated genetic-trait approach identified allowed the authors to link patterns of genetic change to plant functional responses to changing climates, thereby providing a more direct connection between genomic patterns and fitness-related traits. Promisingly, the approach of Lang et al. could also be used to help infer missing genetic or phenotypic data for historical specimens that are unmeasurable, broadening the utility of historical genetic data in poorly sampled regions or taxa.

Finally, one exciting and emerging application for historical genetic data supplied by herbarium collections is its potential use in validating predictions of future adaptive responses (Eckert et al. 2025b). To predict the ability of species to persist in place amid ongoing climate change, researchers often use genotype-environment associations (GEAs) to estimate the degree of (mal)adaptation to future climates (Capblancq et al. 2020) and inform specific management actions like assisted migration or gene flow (Rellstab et al. 2021). Although GEAs are growing in popularity, thanks in part to increases in the availability and accessibility of genetic data, validating future predictions is understandably challenging and rarely performed (Lind and Lotterhos 2024). One way to validate GEA accuracy could be to validate model hindcasts against historical genetic data obtained from herbarium specimens (Eckert et al. 2025b). Similar hindcasting validation approaches have been used to validate species distribution models (Hodel et al. 2022); however, no study to our knowledge has validated GEAs using historical data. Given the extent and coverage of the historical populations reconstructed using our approach, we speculate that hindcast validation of GEAs could be a widely applicable method to test GEA accuracy and gauge the utility of these models for informing conservation. In sum, although we showcase the potential utility of Earth’s herbarium collections for genetic monitoring, the true value of the genetic data housed in Earth’s herbaria extends much further and has broad applications across many fields of ecology and evolution.

## Implications for herbaria, researchers, policy makers, and the public

Our assessment suggests that genetic data housed in Earth’s herbaria likely represents a vast and largely untapped resource for monitoring species genetic response to past and present global change (Eckert et al. 2025b). For herbaria, this means that ensuring the preservation and accessibility of the preserved genetic material housed in their collections is paramount and could influence the success of global genetic monitoring initiatives (Viveiros-Moniz et al. 2025). Our results show that specimens that might have traditionally been considered redundant, such as those originating from the same population, might be of considerable value when it comes to supplying the population-level genetic data needed for monitoring. In line with other applications (Meineke et al. 2018b, Heberling 2022, Mandrioli 2023, Eckert et al. 2024, Jacobs and Griebeler 2025), our work also indicates that specimen digitization efforts are key in unlocking the genetic utility of these collections by providing researchers with knowledge on the extent of available preserved material. As such, we urge curators to consider genetic monitoring applications when setting digitization priorities, making curational decisions (specimens transfer or destruction), or designing strategies for contemporary collection. Alongside specimen preservation and digitization, active collection initiatives are still highly important for addressing remaining spatial and taxonomic gaps in representation (e.g., Canadian Museum of Nature 2024), and resampling of populations preserved in the herbarium record can add additional timepoints that allow us to detect and monitor genetic change.

For researchers, our results suggest herbarium collections can supply the population level data needed to robustly detect and attribute genetic change across thousands of species—opening the door to more broadscale tests of novel and longstanding ecological and evolutionary hypotheses (Meineke et al. 2018b). New work by Vörös and colleagues (2026) also demonstrates how genetic data obtained from herbaria can be integrated alongside data from more classical genetic monitoring programs to extend the temporal scope and scale of assessments of genetic change. However, although some studies have already taken advantage of the genetic data stored in these collections (Eckert et al. 2025b), our analysis indicates that this resource remains largely untapped. Moving forward, we encourage researchers to leverage herbaria as a source of irreplaceable and unique biodiversity data while following best practices (Davis et al. 2024). We stress that when it comes to transforming specimens into genetic EBVs, various considerations will ultimately determine the utility and potential application of herbarium collections. To that end, we discuss some of these considerations and point readers to additional resources in box 3 and in our extended discussion (see Supplemental Section 3).

For policy makers, we reiterate past calls for increased funding to herbaria to facilitate the maintenance and digitization of these valuable collections into the future (Paton et al. 2020, Eckert et al. 2024). Importantly, alongside increasing their value for biodiversity science, empowering herbaria also confers other benefits—herbaria are one of the last refuges for the study of plant taxonomy and systematics (Davis 2023), fields that have experienced declines in funding and representation at institutions around the world (Wheeler 2014). Alongside their scientific value, institutions like herbaria also act as important bridges, connecting the scientific community to other disciplines like the humanities (Jacobs and Griebeler 2025) and the public (Wen et al. 2015), and have important roles to play when it comes to reconciling our colonial past (Park et al. 2023).

Finally, for community science, initiatives informed by existing collections can directly benefit the utility and application of natural history collections to applications like genetic monitoring. For example, Blitz the Gap (blitzthegap.org), a pan-Canadian initiative to address biodiversity data gaps by leveraging public participation through iNaturalist, encouraged participants (by providing priority maps and species lists) to record observations of species captured in the historical herbarium record but not since—thereby increasing our understanding of species persistence across Canada. These sorts of initiatives can directly benefit genetic monitoring applications by helping inform contemporary resampling programs to gather data that complements our natural history collections. In addition, recent work by Panchen and colleagues (2025) highlights how volunteers like community scientists can contribute to projects utilizing herbarium specimens. The authors trained volunteers to extract functional trait (phenological state) information from digitized specimens to enable assessment of phenological change across the Canadian Arctic. Similarly, researchers could use volunteers or community scientists to assist in screening the genetic potential of specimens for genetic analysis, gauging the amount of physical material, corroborating date or locality data, or even traveling to old collecting locations to verify species persistence for contemporary sampling. As the purview of community scientists continues to expand and data housed in herbaria becomes increasingly accessible and mobilized, these historic institutions will become increasingly central to our efforts to understand past and present biodiversity change in the Anthropocene (Meineke et al. 2018b, 2018a).

Box 3. **Limitations and considerations for transforming specimens into genetic EBVs.**

Although our results suggest that herbarium collections can contribute to the genetic monitoring of tens of thousands of species, transforming specimens into genetic EBVs is highly context dependent and involves many considerations (Viveiros-Moniz et al. 2025). In all cases, species and region specific knowledge (e.g., life history traits and landscape connectivity) should be consulted to inform the limits used to delineate populations in the herbarium record—our approach, which was designed to assess the potential contribution of herbarium collections in general to the application of genetic monitoring, may not be appropriate for specific populations, species, or regions (see Supplemental Section 3.1). In general, collaborating with experts on specific flora and consulting available literature can help ensure the limits used are biologically realistic. Importantly, the validity of assigned spatial and temporal limits can also be assessed through post-hoc genetic analysis (Lawson and Falush 2012). Once biologically realistic population limits are determined, our clustering approach can be used to analyze digitally available records, reconstruct populations, and identify candidate specimens. These populations and the spatiotemporal distribution of the specimens they contain should be carefully inspected alongside other species occurrence data and landscape models to maximize the likelihood of panmixia (see Supplemental Section 3.2; Charlesworth and Charlesworth 2010).

The next step in transforming species into genetic EBVs is to contact the herbaria that house candidate specimens. Contacting herbaria can sometimes reveal additional specimens that are undigitized, potentially increasing the sample size of reconstructed populations or even revealing new ones. Herbaria can also offer expertise in validating population limits, verifying specimen metadata (including taxonomic identity and locality), identifying duplicate records, and ultimately, obtaining destructive samples of relevant specimens (see Supplemental Section 3.3).

Extracting and sequencing DNA from herbarium specimens can be more difficult than from contemporary samples due to specimen degradation (Marinček et al. 2022), though novel workflows have mostly overcome this challenge (Bakker et al. 2020, Gouker et al. 2023) and recent reviews highlight this progress (Záveská Drábková 2021, Burbano and Gutaker 2023). Critically, the quality of extracted DNA can vary substantially, influenced by factors like specimen age, available material, and preservation methods (Záveská Drábková 2021, Marinček et al. 2022, Burbano and Gutaker 2023). After DNA is extracted, choice of sequencing will depend on project aims and feasibility and will ultimately determine which metrics of genetic EBVs can be quantified (Papalini et al. 2023). Some targeted sequencing approaches (like amplifying specific loci) tend to produce more reliable genetic data compared to whole genome approaches that often require higher DNA quality and reference genomes for accurate sequence read alignment (McCormack et al. 2017). Sequencing approach will also affect interpretation of the derived metrics. For instance, metrics that focus on adaptive diversity can respond faster to factors like environmental change, whereas metrics computed from mostly neutral markers can remain relatively stable over the same time period (see Supplemental Section 3.4; Wright 1931, Kimura 1962, Chung et al. 2023). Indeed, targeting specific genetic regions that govern known phenotypic responses to environmental change can help detect significant genetic signals in typically noisy genomic data (Lang et al. 2024, Whiting et al. 2024).

Once EBVs have been estimated, researchers should report and publish these estimates to contribute to global monitoring efforts (see Supplemental Section 3.5). Methodology, protocols, all genetic data, including raw sequences, and metadata should be archived in public repositories like GenBank or GEOME (Deck et al. 2017). These steps are critical as poor data archiving and lack of georeferenced sequences remain major obstacles to improving our understanding of genetic change globally (Toczydlowski et al. 2021). Importantly, standardized and complete archiving also allows for data from multiple studies to feed into high-level biodiversity monitoring frameworks like the Group on Earth Observations Biodiversity Observation Network’s (GEO BON) EBV cube (Jetz et al. 2019).

These and additional considerations are discussed at greater length in the Supplementary Material (Section 3), where we also point readers to relevant reviews and best practices (table S11).

## Realizing the potential of natural history collections requires a collaborative approach

Ultimately, our ability to transform herbarium specimens into the data we need for standardized genetic monitoring will depend on communication and collaboration across researchers, institutions, and nations. Around the world, there are around 4000 active herbaria (Thiers 2024) housing the collections that, only when combined across institutions, are able to realize their full potential. To reconstruct populations, our results show that we typically need specimens from 3 to 4 different herbaria, with some populations being spread across as many as 25. Thus, obtaining the knowledge, resources, and physical samples required to mobilize the genetic data housed in Earth’s herbaria will likely necessitate collaboration across researchers, institutions, and nations.

Luckily, herbaria are no strangers to collaboration. Around the world, many institutions have self-organized into networks that share resources, skills, and knowledge (Beaman 1965). For example, efforts since 2007 to aggregate digital collections under the *Canadensys* (www.canadensys.net) network have contributed to the digitization of over 900 K herbarium specimens in Canada and produced a wealth of shared knowledge and technology. Supporting initiatives like *Canadensys*, in addition to preserving expert skills and knowledge, can also benefit digitization efforts, helping transform herbaria into sources of critical biodiversity data (Eckert et al. 2024) and hubs for global change research (Meineke et al. 2018b).

To this end, the vision of an open access *global metaherbarium* has been proposed to standardize, facilitate, and streamline the digitization of *extended specimens*, complemented by layers of additional functional, genetic, and ecological data (Lendemer et al. 2020). Similarly, the emerging field of *collectomics* proposes digital frameworks that can support all current and future data and knowledge obtained from specimens (Sigwart et al. 2025), and existing projects like iDigBio (www.idigbio.org) have made significant progress in developing fully integrated, digitized, and accessible natural history collections. Recent work also highlights the potential for leveraging public involvement (Ellwood et al. 2015, Panchen et al. 2025) and artificial intelligence (Stenhouse et al. 2025) to aid digitization efforts. Empowering researchers, herbaria, and the networks they comprise would help accelerate their digital transition and fast-track the mobilization of the valuable biodiversity data housed in these collections (Eckert et al. 2024).

## The growing role of herbaria in genetic monitoring, research, and conservation

Amid accelerating global change (Ripple et al. 2025), the Kunming-Montreal Global Biodiversity Framework solidified humanity’s commitment to preventing biodiversity loss and the erosion of critical ecosystem services (United Nations Convention on Biological Diversity 2022). Core to this framework, and to conservation in general, is the maintenance of genetic composition (Lande 1995, Kardos et al. 2021). However, efforts to monitor genetic change across many species and regions are limited (Hoban et al. 2021), largely due to a lack of available data. Here, we report that the genetic material housed in Earth’s herbaria could help shrink global data gaps for vascular plants. Unlike the monitoring of other kingdoms, this vast trove of historical specimens offers an unparalleled view of population-level genetic composition through time. Mobilizing this genetic data would transform our ability to detect and attribute genetic change, provide critical baselines to empower contemporary monitoring programs, and ultimately, benefit conservation efforts (Viveiros-Moniz et al. 2025). Given their ability to supply critical biodiversity data and contribute to overcoming global data shortfalls, natural history institutions like herbaria continue to play a key role in our understanding of biodiversity and our ability to protect it.

## Supplementary Material

biag048_Supplemental_File

## Data Availability

A full list of all the reconstructed populations, a tool to visualize them, as well as code to reproduce the analysis and make the figures is available in the Figshare repository: 10.6084/m9.figshare.27198381.

## Author Biography

Isaac Eckert (isaac.eckert@mail.mcgill.ca), Lucas Eckert, Olivia Rahn, and Cameron So are doctoral students at McGill University in Montréal, Québec, Canada. Isaac Eckert works on transforming novel and traditional sources of biodiversity data into ecological knowledge to inform conservation, Lucas Eckert aims to understand the patterns and processes of rapid evolution, Olivia Rahn studies the ecological drivers of plant range limits, and Cameron So is working to resolve genomic patterns and processes across plant ranges. Together they have published past work in the field of herbarium genetics, including how we can harness historical specimens to understand genetic change in the Anthropocene. Laura J. Pollock is an associate professor in the McGill University, Montréal, Québec, Canada. Laura J. Pollock leads a research group that works to model and monitor biodiversity change to inform systematic conservation planning. Simon Joly is an adjunct professor at Université de Montréal, Montréal, Québec, Canada, and a botanist and researcher at the Jardin Botanique de Montréal. Simon Joly’s research group studies the evolution of plants using ecological, genetic, and phylogenetic approaches, often leveraging herbarium collections. All authors are members of the Québec Centre for Biodiversity Science, Montréal, Québec, Canada.
