# Supplementary material for: The utility of herbarium collections for genetic monitoring
Source: Bioscience. 2026 May 6;76(9):787–800. doi: 10.1093/biosci/biag048 (PMC13572878; doi:10.1093/biosci/biag048)
Supplement: Supplementary file 1 [file biag048_supplemental_file.docx]

**TABLE OF CONTENTS:**

Section 1) Supplementary Methods – Page 1

Section 2) Supplementary Results – Page 6

Section 3) Supplementary Discussion – Page 6

Section 4) Supplementary Tables – Page 8

Section 5) Supplementary Figures – Page 14

Section 6) Supplementary References – Page 16

**Section 1) SUPPLEMENTARY METHODS**

**1.1 Overview**

For herbaria to provide the genetic data needed to quantify population-level genetic EBVs, specimens need to represent historical plant populations. We define these historical *populations* as groups of specimens from the same species that were collected from the same geographic area, during the same period, and that meet or exceed the minimum sample sizes required to estimate genetic EBVs (Table 1). To identify populations, we first downloaded digitized herbarium records from the Global Biodiversity Information Facility (GBIF; Fig. 2a) and identified species-specific spatial and temporal population limits based on a subset of functional traits (Fig. 2b). These limits were used to assemble historical populations by linking specimens in space and time (Fig. 2c) and recording populations that met minimum sample size criteria (Fig. 2d). In the case of genetic differentiation, we identified populations that were spatially distinct but sampled in the same period that would allow for a snapshot of between population differentiation (Fig. 2e). Once populations were identified, we used locality data from the World Checklist of Vascular Plants (WCVP; Govaerts et al., 2021) to assign occurrence status (Fig. 2f) before assembling populations with repeated temporal sampling into time series (Fig. 2g-h). Finally, we summarized for each species the extent to which digitized herbarium specimens could be used to quantify snapshots and series of genetic EBVs (Fig. 2i) to assess spatial, temporal, and taxonomic coverage.

**1.2 Downloading GBIF data**

To download digitized herbarium specimens hosted on GBIF, we used the “rgbif” package for R (Chamberlain et al., 2024). Specifically, we specified that the phylum must be Tracheophyta (vascular plants) using the phylum key 7707728. We also specified that the “basisOfRecord” must be “PRESERVED_SPECIMEN”, that “hasCoordinate” must be “TRUE”, and that “hasGeospatialIssue” must be “FALSE”. After downloading, we also aggregate records to the species level by removing records that were not identified to at least species and aggregating those identified beneath species (e.g., variety, form, subspecies) to their corresponding species. Finally, we cleaned records by removing all records that fell within 500m of academic institutions and herbaria using the coordinate cleaner package for R (Zizka et al., 2019). Recorders were accessed in October of 2024 (GBIF.Org, 2024).

**1.3 Defining a population: Estimating geographic and temporal thresholds**

Past macrogenetic studies often employ unrealistic delineations of populations, which can hinder the relevance and application of their results. Paz-Vinas et al. (2021) suggest using more realistic cutoffs to define population units, potentially informed by species functional traits. Since genetic diversity can vary both spatially and temporally, we attempted to define geographic and temporal boundaries for populations. To define the geographic limits of potential plant populations, we used a dataset from Lososová et al. (2023) which estimates dispersal classes for over 10K plant species using a combination of empirical data and models informed by plant height, dispersal syndrome, growth form, habitat, and diaspore features. For species represented in this dataset, we simply used the corresponding dispersal class. If a species was not represented in the dataset, but its genus was, we set its dispersal class to the genera average. If the genus was not represented, we set the dispersal class to the family average. For species whose families were not in the dispersal dataset, we instead used a family level estimate of growth form and the average dispersal class for that growth form to assign a dispersal class. To limit the uncertainty associated with assigning dispersal class in this way, we aggregated the 6 initial dispersal classes described in the dataset to just 3 (poor, moderate, and good dispersers). Finally, we assigned each of these classes a geographic distance threshold to delineate populations. For poor dispersers we used a maximum distance of 1km between records, for moderate dispersers we used 5km, and for good dispersers we used 15km. Compared to past plant macrogenetic studies, these spatial population constraints are considerably more conservative (Millette et al., 2020).

We recognize that plant populations also exchange genetic material via pollen (Dick et al., 2008; Slarkin, 1985), and that delineating populations by the extent of pollen exchange could be biologically relevant. However, past work suggests that pollen dispersal often operates on larger spatial scales compared to seed dispersal (Bacles and Ennos, 2008), meaning that our approach of using seed dispersal is likely more spatially conservative. For tree species, which have some of our broadest spatial clustering constraints, there is empirical evidence supporting the idea that gene or pollen flow from a focal population can occur over distances of tens to hundreds of kilometres (Savolainen et al., 2007). While this might suggest that our limits are overly restrictive, we argue that smaller spatial limits increase the comparability of samples from multiple time points, and therefore, should be preferred for these types of macrogenetic studies. Additionally, because of our clustering method (see section below), two individual samples in a population might be separated by a distance greater than this limit, providing another reason to choose restrictive values.

We used estimates of generation time to set species specific temporal population thresholds, as we know that generation time is a predictor of the speed of genetic change in populations (Lehtonen and Lanfear, 2014; Pollak, 1982; Smith and Donoghue, 2008). Data from Compagnoni et al. (2021) shows that growth form can be used to predict generation time, and thus we used growth form to categorize species as having either “short” or “long generation” times, along with an additional 1-year category for annual plants identified using the WCVP. We considered temporal clusters to be 1 year, 2 years, and 5 years for annual, short-generation time, and long-generation time species respectively. Again, these limits might seem excessively restrictive; for example, genetic change in long-lived tree populations is unlikely even at time scales greater than 5 years. However, we once again argue that by making limits more restrictive, we increase the comparability of samples at different time points.

**1.4 Clustering historical herbarium specimens into populations**

For each species, we identified sampled historical populations by grouping records using a stepwise clustering approach. First, we calculated the pairwise distance between all records in geographic and temporal space. From these distance matrices, we isolated pairs of records that fell within our geographic and temporal population thresholds (See Fig. S1). To calculate geographic distance, we used the “geodist” package for R and the “cheap” distance function which allows for fast and accurate calculations of distance across at smaller (<100km) spatial scales (Padgham and Sumner, 2024). This produced a list of “linked” records that could be within the same population. Then, starting with the record with the most “links”, we assigned that focal record, and all its linked records a cluster identifier (e.g., 1). The records in this cluster were then removed from the list, and the procedure was repeated until there were no records left to assign to a cluster. Finally, we removed clusters that did not meet the minimum sample size requirements. This approach differs from other clustering approaches in that it attempts to maximize sample size within clusters – ensuring that the identified populations contain as many potential individuals as possible. Because the direction of the “links” between records is not considered, our thresholds used to define populations are better thought of as radii extending from the focal record of each cluster in both the spatial and temporal planes. For example, if a focal record was collected in the year 2020, then, assuming that species is an annual with a temporal threshold of 1 year, that population can only contain records from 2019, 2020, or 2021. If that species had a temporal threshold of 5 years, then the population could contain records from 2015-2025. While we provide clustering method that generally creates biologically meaningful populations, we recognize that other clustering methods might result in different clustering of specimens that are not necessarily less valid. In future studies of specific species and regions, we would encourage researchers to attempt multiple clustering approaches to determine which is most appropriate for their data and aims.

**1.5 The challenge of Genetic Differentiation**

Genetic Differentiation is a pairwise metric and thus requires at least 2 populations to quantify. To get a single snapshot of Genetic Differentiation in time, we needed to identify species with sampled populations that were spatially distinct (i.e. the geographic centroids of the populations are separated in space by at least the species-specific spatial population threshold) but were sampled in the same time period (i.e. the mean sampling years for each population are separated in time by less than the species-specific temporal population threshold). To do this we used a pairwise approach, identifying per species the combinations of populations that satisfied these criteria. Because this approach is pairwise, a single sampled historical population can contribute to many potential measures of Genetic Differentiation – leading to many potential snapshots across all species. Therefore, our estimates for the number of potential measures of Genetic Differentiation were frequently much higher than that of the other genetic EBVs we assess in this paper.

**1.6 Note on duplication:**

It is not uncommon for herbarium collectors to make duplicate records from the same individual plant for taxonomic purposes. These duplicates are often shared between institutions to serve as local type specimens. Because you need abundant material to make copies, this practice is most common in larger plants like trees, shrubs, and grasses. The inclusion of duplicated records in our identified populations would be problematic for genetic analyses which aim to capture variation between individuals in a population. We tried to identify as many duplicate records as possible using a combination of comparing collection locations and dates, collector names, and verbatim record numbers; however, this approach is limited by the extent to which that information is available on GBIF. As such, we highlight the importance of working with herbarium workers, taxonomic experts, and collectors to inspect the individual specimens to identity and remove duplicates before conducting genetic analyses. In our experience, it is usually easy to identify duplicates once you can view the physical specimens and read additional information such as descriptions or notes that are often not digitized.

Conversely, it is common for single herbarium records (sheets) of smaller plants to contain more than one individual. Or for herbaria to thin the number of records down, to prioritize spatially and temporally distinct records rather than replicates in the digitization process. Both practises could increase the number of individuals available for sampling for genetic analysis, potentially balancing the negative impacts of record duplication in some cases. Nonetheless, these specific challenges and opportunities highlight the complexities of working with historical collections and we suggest that researchers leveraging these preserved specimens consider these nuances to ensure production of robust and scientifically valid genetic data.

**1.7 Assigning occurrence status (native, introduced, extinct)**

To identify whether populations occurred in the native plant range, we used the plant distributions as part of the WCVP and the Level 3 botanical country delineations provided through the “rWVCP” package for R (Brown et al., 2023). For most plant species, WCVP provides information on which botanical countries the species is considered native, introduced, or extinct. To assign occurrence status to our identified clusters, we first identified which botanical country the cluster occurred in by intersecting the botanical country polygons with our clusters using the “terra” package for R (Hijmans et al., 2022). Next, we cross referenced the botanical countries against the WCVP database to assign either native, introduced, or extinct status. When a cluster fell outside of the delineated botanical countries (which rarely occurred but did affect some small oceanic islands) we assigned it to the nearest botanical country based on geographic distance. If the assigned botanical country was not listed as part of a species native, introduced, or extinct range, we again assigned it the occurrence status of the nearest in-range botanical country based on geographic distance.

**1.8 Fitting accumulation curves**

To understand how the digitization of additional specimens might enable the implementation of our proposed genetic EBV workflows, we used modelled accumulation curves. During the clustering analysis, when records were assigned to a successful population, we made sure to record the GBIF occurrence ID. After running the analysis for all species, we were left with a list of all GBIF records that contributed to producing a historical population. Using these IDs and the population clusters these records were assigned to; we built random accumulation curves. The process involved randomizing the order of all GBIF records, before accumulating records in sequence, while recording the number of population clusters, the number of temporally replicated clusters, as well as the number of species those accumulated clusters responded to. Because we analyzed over 50M GBIF herbarium records, we accumulated records in blocks of 100K to reduce wall time. The entire accumulation analysis was repeated 1000 times to produce a distribution, which was used to fit models to estimate the rate of accumulation. To model the accumulation of species with population clusters, we needed to use models that account for the fact that there is a finite number of vascular plant species on Earth. As such, following past work (Eckert et al., 2024), we used zero-inflated beta regressions. Beta regressions work by modelling data between 0 and 1, assuming natural asymptotes at those values. Zero-inflated beta regressions can also incorporate 0s allowing us to include all our accumulation data (usually it took a few hundred thousand records to accumulate a single species with a population cluster). To use these beta regressions, we first transformed the number of species accumulated into a portion of all species, ranging from 0 to 1, and log-transforming the number of records. In general, Beta Regression models fit the data extremely well, even when including all 1000 randomizations in the model fitting process (see Tables S3-8).

**1.9 Extrapolating curves**

Since only 21% (~83316867) of Earth’s 396746986 herbarium specimens are digitized, that leaves an additional ~313M specimens left to digitize. Between institutional quality control, GBIF’s filtering steps, and filtering to only vascular plants, of the 83M digitized specimens, only 51,230,881 (61.5%) were retained for our analysis. Assuming this percentage stays the same, that means roughly 192,725,704 undigitized specimens could be available for analyses such as ours. To understand how these additional specimens might enable the operationalization of genetic EBVs we extrapolated our accumulation curves to an additional 192,725,704 to estimate the number of additional populations, the number of temporal replicates, and the number of represented species, potentially housed in these undigitized specimens.

**1.10 Evaluating historical population persistence**

Contemporary sampling of genetic data can be used to add an addition time point to populations that have been historically sampled, thereby turning potential snapshots into potential time series. However, plant populations are highly dynamic, and many historical populations may have not persisted through time – limiting our ability to sample them in present day. Thus, we needed to identify which populations have likely persisted and are available to be sampled. To do this we used iNaturalist data and recent herbarium records. iNaturalist is a commonly used community science platform that has produced millions of research-grade observations over the past decade, offering unparalleled resolution of contemporary biodiversity.

To start, we downloaded all research grade iNaturalist observation in the phylum *Tracheophyta* from GBIF using the rgbif package for R*.* We specified only iNaturalist observations by only accessing GBIF records in the dataset with the following key: “50c9509d-22c7-4a22-a47d-8c48425ef4a7”. We also excluded records with geospatial issues and those without coordinates.

To relate iNaturalist observations to sampled historical populations of herbarium specimens, we used the following procedure. For each species, we first gathered all synonyms from the WCVP database, before isolating all iNaturalist observation corresponding to that species (and its synonyms). Then we transformed both the sampled historical populations corresponding to that species and the iNaturalist records into spatial points. We then buffered the sampled historical populations by the species-specific population distance threshold before intersecting those historical polygons with spatial iNaturalist records using the *relate* command in the terra package for R. If an iNaturalist record intersected with the buffered historical population, we recorded the year that record was observed. After all intersections were identified, we aggregated the resulting data by year, retaining the most recently observed record. Finally, the year of that record was added to the historical population data and represents the most recent year an individual of that population was recorded. This was repeated for all species and for all historical populations.

We considered historical populations as candidates for contemporary sampling if (1) there was a recent (2014-2024) herbarium specimen from that population or (2) there was a recent (2014-2024) record of that population in iNaturalist. However, we expect that this is likely an underestimation of population persistence; many populations may have persisted but have not been observed in the last decade, which would not be surprising given the patchiness of iNaturalist observations. Therefore, we believe this is a conservative estimate of the potential for contemporary sampling.

**Section 2) SUPPLEMENTARY RESULTS**

**2.1 Antarctica**

According to the WCVP, Antarctica contains only 3 native species of vascular plant (*Deschampsia antarctica*, *Deschampsia parvula*, and *Colobanthus quitensis*). Of these, *D.* *antarctica* and *C. quitensis* have historical populations that can be used to a snapshot of quantify genetic diversity. Only for *D.* *antarctica* which has 2 populations dating to 1962 and 1964, it is possible to quantify a snapshot of genetic differentiation for that period. The single population representing *C. quitensis* is from 1964. No measures of effective population size or inbreeding are possible for any species. All populations were collected in the area around the British-operated Signy Research Station on Signy Island which is a part of the South Orkney Islands which are roughly 600km offshore of the Antarctic Peninsula.

**2.2 Series**

Recent meta-analytical attempts to monitor change in genetic diversity across the tree of life revealed widespread declines (Shaw et al., 2025), however their analysis was limited by available time-series length (median = 6 years), time period captured (median midpoint = 2000), and number of timepoints (94% of the data was a series of 2 timepoints). While most *series* we identified in herbarium collections only contain 2 timepoints, they capture considerably longer timespans (median = 18, 20, & 15 for genetic diversity, differentiation, and inbreeding and effective population size respectively), better represent historical time periods (median midpoint = 1967 for genetic diversity and differentiation and 1948 for inbreeding and effective population size), and we identified thousands of potential *series* with 3 or more timepoints (Table 2).

**Section 3) SUPPLEMENTARY DISCUSSION**

**3.1 Species specific considerations**

Starting with a species and region of interest, existing literature, naturalist knowledge, and available functional trait data (e.g. mating system, dispersal syndrome, longevity) should be consulted to inform the limits used to delineate populations in the herbarium record. For example, in well-connected ecosystems, populations can span much larger geographic distances than the limits used in this study (Kremer et al., 2012). Conversely, smaller geographic limits may be required to accurately delineate populations in disconnected landscapes such as alpine ecosystems or fragmented habitat. Landscape connectivity can also interact with pollination syndrome to determine gene flow and population differentiation (Hughes et al., 2007) and different pollination syndromes can also influence spatial genetic patterns (Kramer et al., 2011), all of which should be considered when setting population limits for specific taxa. Variation in mating systems (e.g. clonality) also warrants additional considerations like choosing appropriate genetic markers or ensuring that herbarium specimens represent different genets (Arnaud-Haond et al., 2005).

**3.2 Ensuring panmixia**

When using genetic metrics (like genetic EBVs) based on Hardy–Weinberg equilibria, researchers usually assume the genetic data that they cluster into populations come from panmictic populations and represent individuals with random (or equal opportunity) mating. While many studies use population-level genetic data from herbarium specimens, few discuss whether this data is likely panmictic. We tried to design our methodology to be conservative in this fashion, trying to ensure that the specimens we identified could represent panmictic population, but ultimately, we are relying on an assumption. Luckily, post-hoc approaches of evaluating population structure, panmixia, and identifying panmictic subpopulations exist and would be well suited to use with herbarium data (Evanno et al., 2005).

**3.3 Working with herbaria**

After spatial and temporal limits are determined, our approach can be used to identify historical populations and candidate specimens. Contacting herbaria can sometimes reveal additional specimens that are undigitized but can be used to represent historical populations. Additionally, some herbaria curate DNA banks which store preserved tissues or raw DNA extracts, although the latter are relatively rare. For example, the New York Botanical Garden curates a DNA bank of over 25K tissue samples, of which 500 have corresponding DNA extracts (Hodkinson et al., 2007). Taking advantage of these samples, when they exist, can help reduce the costs of transforming specimens into genetic EBVs. Herbaria curators, workers, and collectors should be consulted throughout the process to help verify specimen metadata (including taxonomic identity and locality), identify potentially duplicated records, and ultimately obtain destructive samples of relevant specimens. For best practices in working with herbaria and their specimens, see Davis *et al.* (2024).

**3.4 Sequencing and metrics**

For DNA sequencing, researchers should review project feasibility (e.g budget, sample sizes, DNA quality) before choosing a sequencing platform for nuclear DNA. Given generally reduced costs in recent years, reduced representation sequencing (e.g RAD-seq) and whole genome sequencing (e.g low-coverage WGS) has become more popular among researchers and is forward compatible with future studies. In other cases, researchers may rely on older techniques such as microsatellites if previous workflows are already established (e.g Shi, 2004). Some species such as conifers have large genomes (>1Gb) and are generally sequenced with exome capture to reduce costs and target only protein-coding regions (Neves et al., 2013). Overall, each project will require consideration of the goals, study-species, and feasibility. We direct researchers to recent reviews for different sequencing approaches (Andrews et al., 2016; Lou et al., 2021). When sequencing is completed, raw reads must be aligned with a reference or *de novo* reference, quality controlled and filtered before conducting downstream analyses.

Careful consideration should also go into the metrics that are investigated (many examples of potential metrics and tools for estimating EBVs are reported in Table S1 and in Hoban et al. (2022). Neutral metrics such as genetic diversity (π and H_o_) and genetic differentiation (F_ST_) may show marginal changes even over multiple generations because the rare alleles first lost to drift contribute little to these metrics (Kimura, 1962; Wright, 1931). This is especially true for species with long generation times such as trees (Lowe et al., 2005). As an alternative, researchers could prioritize populations expected to respond to environmental change and where the genetic basis of these responses is known (Chung et al., 2023; Willi et al., 2022). For instance, flowering phenology is commonly known to to adaptively evolve to climate warming (Anderson et al., 2012; Franks et al., 2007) and given the genetic basis has been discovered for some species (Yan et al., 2021), researchers could monitor specific populations and loci where rapid evolution is predicted. Excitingly, Whiting et al. (2024) found 108 gene families and gene functions repeatedly driving local adaptation to climate in 25 diverse plant species. An interesting yet untested avenue is to identify similar gene families and allele frequency changes in other non-model plant systems.

**3.5 Data archiving**

For best practices see Waples *et al.* (2018).

**Section 4) SUPPLEMENTARY TABLES**

**Table S1** — Anova of empirically estimated generation length and growth form. To achieve normality, generation length was log-transformed.

| **Variable** | **df** | **Sum Sq** | **Mean Sq** | **F value** | **p** |
| --- | --- | --- | --- | --- | --- |
| Growth Form | 7 | 305.5 | 43.64 | 25.02 | <0.001 |
| Residuals | 337 | 587.7 | 1.74 |  |  |

**Table S2** — T-test between short and long generation time groups and empirical generation time estimates. To achieve normality, generation length was log-transformed. Mean generation time of “short” lived plants was 11.3 years and mean of “long” lived plants was 63.0 years.

| **t** | **df** | **p** |
| --- | --- | --- |
| - 14.163 | 407.84 | <0.001 |

**Table S3** — Model estimates for the relationship of accumulation of species for which we have snapshots (at least 1 sampled historical population) of **genetic diversity** over the index of herbarium records. These accumulation curves were built by randomizing the order of all herbarium specimens, before accumulating theme in chunks of 100000, where after each accumulation, we recorded the number of species with sampled historical populations that met or exceeded minimum sample size requirements. This process of simulating “random” accumulation was repeated 1000 times to get a distribution which was used to fit a Generalized Additive Model for Location Scale and Shape (GAMLSS). This model estimates the effect of a predictor on a response variable that varies between 0 and 1 and so we transformed species richness into the portion of total species. By using a Zero-Inflated Beta Distribution, we were able to include the first few data points where the number of species accumulated was often 0. We log transformed our independent variable (number of herbarium specimens) which allowed for very good model fit. Model AIC = - 5718538 and R^2^ = 0.999 with 513000 observations and 512996 residual degrees of freedom.

| **Parameter** | **Link** | **Estimate** | **Std. Error** | **t** | **p** |
| --- | --- | --- | --- | --- | --- |
| Mu-Intercept  **Mu-Slope** | Logit | -12.21  **1.638** | 0.0005902  **0.0001009** | -20683  **16239** | <0.001  **<0.001** |
| Sigma-Intercept | Log | 10.230894 | 0.001971 | 5190 | <0.001 |
| Nu-Intercept | Logit | -6.34834 | 0.03344 | -189.9 | <0.001 |

**Table S4** — Model estimates for the relationship of accumulation of species for which we have snapshots (at least 1 sampled historical population) of **genetic differentiation** over the index of herbarium records. The model is described above in Table SX. Model AIC = - 7158946 and R^2^ = 0.999 with 513000 observations and 512996 residual degrees of freedom.

| **Parameter** | **Link** | **Estimate** | **Std. Error** | **t** | **p** |
| --- | --- | --- | --- | --- | --- |
| Mu-Intercept  **Mu-Slope** | Logit | -14.68  **1.843** | 0.0004755  **0.0000807** | - 30881  **22837** | <0.001  **<0.001** |
| Sigma-Intercept | Log | 12.043508 | 0.001984 | 6070 | <0.001 |
| Nu-Intercept | Logit | - 4.41721 | 0.01286 | - 343.4 | <0.001 |

**Table S5** — Model estimates for the relationship of accumulation of species for which we have snapshots (at least 1 sampled historical population) of **inbreeding** and **effective population size** over the index of herbarium records. The model is described above in Table SX. Model AIC = -7708398 and R^2^ = 0.998 with 513000 observations and 512996 residual degrees of freedom.

| **Parameter** | **Link** | **Estimate** | **Std. Error** | **t** | **p** |
| --- | --- | --- | --- | --- | --- |
| Mu-Intercept  **Mu-Slope** | Logit | -15.61  **1.786** | 0.0009750  **0.0001655** | -16012  **10791** | <0.001  **<0.001** |
| Sigma-Intercept | Log | 11.801904 | 0.001975 | 5976 | <0.001 |
| Nu-Intercept | Logit | -4.66605 | 0.01453 | -321.2 | <0.001 |

**Table S6** — Model estimates for the relationship of accumulation of species for which we have a series (n=2) of **genetic diversity** over the index of herbarium records. The model is described above in Table SX. To model series accumulation we increased the maximum number of fitting iterations using the Rigby and Stasinopoulos algorithm from 20 to 50. Model AIC = -7788201 and R^2^ = 0.999 with 513000 observations and 512996 residual degrees of freedom.

| **Parameter** | **Link** | **Estimate** | **Std. Error** | **t** | **p** |
| --- | --- | --- | --- | --- | --- |
| Mu-Intercept  **Mu-Slope** | Logit | -16.60  **2.02** | 0.0005476  **0.0000925** | -30316  **21827** | <0.001  **<0.001** |
| Sigma-Intercept | Log | 12.746045 | 0.001997 | 6381 | <0.001 |
| Nu-Intercept | Logit | -3.661564 | 0.008934 | -409.8 | <0.001 |

**Table S7** — Model estimates for the relationship of accumulation of species for which we have a series (n=2) of **genetic differentiation** over the index of herbarium records. Model AIC = - 7719135 and R^2^ = 0.993 with 513000 observations and 512996 residual degrees of freedom.

| **Parameter** | **Link** | **Estimate** | **Std. Error** | **t** | **p** |
| --- | --- | --- | --- | --- | --- |
| Mu-Intercept  **Mu-Slope** | Logit | -23.12  **2.75** | 0.001992  **0.0003332** | -11604  **8247** | <0.001  **<0.001** |
| Sigma-Intercept | Log | 12.817547 | 0.002102 | 6097 | <0.001 |
| Nu-Intercept | Logit | - 1.938977 | 0.004211 | - 460.5 | <0.001 |

**Table S8** — Model estimates for the relationship of accumulation of species for which we have a series (n=2) of **effective population size** and **inbreeding** over the index of herbarium records. Model AIC = - 8380011 and R^2^ = 0.991 with 513000 observations and 512996 residual degrees of freedom.

| **Parameter** | **Link** | **Estimate** | **Std. Error** | **t** | **p** |
| --- | --- | --- | --- | --- | --- |
| Mu-Intercept  **Mu-Slope** | Logit | -19.60  **2.036** | 0.001764  **0.0009278** | -11117  **6837** | <0.001  **<0.001** |
| Sigma-Intercept | Log | 13.364123 | 0.002083 | 6415 | <0.001 |
| Nu-Intercept | Logit | -2.127135 | 0.004526 | -470 | <0.001 |

**Table S9** — Predictions for the potential number of species with snapshots and series of genetic EBVs under full digitization scenario. Despite fitting our accumulation models on a distribution of 1000 random accumulation simulations, model fit was very good (see above tables) and prediction intervals were narrow. That said, these estimates are a product of the existing data and are not necessarily representative of our undigitized collections. Until we digitize Earth’s remaining specimens, it will remain unclear whether their stored genetic material can be harnessed to fill global gaps in our understanding of plant genetic composition. Note that the large predicted gains in species represented by potential series of genetic differentiation are a result of many individual populations being required to construct these series. Unlike the other EBVs which require 2 populations to build a 2 timepoint series, genetic differentiation needs 4 populations to be able to estimate change in differentiation over time. This caused the accumulation curves to flatten out over low numbers of added records, since the chances of constructing the necessary 4 populations for any one species is relatively low. After enough records are accumulated, the rate at which we accumulate series of genetic differentiation is quite rapid, causing the curve to terminate with a fairly steep slope. This makes sense, since the chances of new records completing a new population that would allow for a new measure of genetic differentiation through time increases dramatically with the number of already added records. This is reflected in the mu (slope) estimates of Table S7.

| **EBV** | **Datatype** | **Current Potential** | **Estimate (portion of species)** | **Portion Increase (estimate / current)** | **Estimate (number of species)** | **Prediction Interval (number of species)** |
| --- | --- | --- | --- | --- | --- | --- |
| Genetic Diversity | Snapshot | 0.117 | 0.638 | 5.45 | 228092 | ±962 |
| Genetic Diversity | Series (n=2) | 0.018 | 0.301 | 16.72 | 107470 | ±105 |
| Genetic Differentiation | Snapshot | 0.039 | 0.423 | 10.85 | 151014 | ± 201 |
| Genetic Differentiation | Series (n=2) | 0.0024 | 0.156 | 64 | 55682 | ± 26 |
| Effective Population Size & Inbreeding | Snapshot | 0.011 | 0.157 | 14.27 | 56075 | ±69 |
| Effective Population Size & Inbreeding | Series (n=2) | 0.001 | 0.024 | 24 | 8424 | ±10 |

**Table S10** — Results from persistence analysis of populations. We defined persisting populations as populations that have been sampled in the last 10 years either by recent herbarium collection or through iNaturalist observations. The resampling of these persistent populations could add an additional timepoint of data, allowing us to generate n=2 timeseries for species that are currently unrepresented. The gains (sampling increase) are moderate compared to the potential gains in digitization since the resampling of populations is dependent on the populations we have already identified in digitized herbarium data. Over time, as we digitize additional specimens, the potential gains from contemporary resampling will increase as more and more populations become available for resampling. Thus, right now, it might make more sense to focus on digitizing remaining specimens to better understand the taxonomic and spatial distributions of historical populations to enable more valuable and targeted contemporary sampling.

| **EBV** | **Number of species with series (n=2)** | **Number of persisting populations** | **Portion of persisting populations** | **Number of species gained via contemporary sampling of persistent populations** | **Sampling increase** | **Total potential portion of species represented** |
| --- | --- | --- | --- | --- | --- | --- |
| Genetic Diversity | 6282 | 43510 | 0.27 | 11578 | 2.8 | 0.050 |
| Genetic Differentiation | 871 | 43510 | 0.27 | 3474 | 5.1 | 0.012 |
| Effective Population Size & Inbreeding | 362 | 3673 | 0.33 | 1500 | 5.2 | 0.005 |

**Table S11** — Existing resources and best practises for transforming herbarium specimens into genetic EBVs.

| **Topic** | **Description** | **Resource** |
| --- | --- | --- |
| Cleaning data from GBIF | Paper discussing the impact of various data cleaning pipelines on downstream modelling applications | (Führding‐Potschkat et al., 2022) |
| Cleaning data from GBIF | Analysis of different cleaning methods using various error rates | (Zizka et al., 2020) |
| Genetic EBVs | Review of genetic EBVs | (Hoban et al., 2022) |
| Current genetic monitoring initiatives | Reviews of the status of genetic monitoring in Europe | (Pearman et al., 2024, 2023) |
| Applying herbarium collections to genetic monitoring | Proof of concept for our idea | (Viveiros-Moniz et al., 2025) |
| Macrogenetics | Considerations in terms of realistic population limits, marker selection, etc. | (Paz-Vinas et al., 2021) |
| Working with herbaria | Logistical, scientific, and ethical considerations | (Davis et al., 2024) |
| Sampling design | Methods for post-hoc assessment of panmixia |  |
| Extracting DNA from herbarium specimens | Various examples and review papers for methods and approaches to ensuring high quality DNA during extraction from herbarium specimens | (Bakker et al., 2016; Bradshaw et al., 2023; Brewer et al., 2019; Brüniche–Olsen et al., 2018; Burbano and Gutaker, 2023; Campos et al., 2023; Latorre et al., 2020; Marinček et al., 2022; Poinar and Cooper, 2000) |
| Sequencing DNA | Reviews talking about sequencing considerations | (Andrews et al., 2016; Lou et al., 2021) |
| Bioinformatics | Approaches to analyze genetic data from herbarium specimens | (Latorre et al., 2020) |
| Choice of genetic metrics | Discussion of genetic metrics and how they relate to genetic EBVs | (Hoban et al., 2022) |
| Integrated trait-genomic approaches | Example using an integrated trait-genomic method | (Lang et al., 2024) |
| Genetic data archiving and analysis | Importance of archiving and best practises | (Deck et al., 2017; Toczydlowski et al., 2021; Waples et al., 2018) |
| Frameworks for integrating genetic data during digitization | Vision and framework for including auxiliary data like genetic data in digital specimens | (Davis, 2023; Lendemer et al., 2020; Sigwart et al., 2025) |

**Section 5) SUPPLEMENTARY FIGURES**

***Figure S1*** —Using expert-informed geographic and temporal population limits, herbarium specimens can be linked together in space (a) and time (b) to increase the likelihood they constitute a panmictic population. When specimens fall beyond these limits, they are considered repetitive of a different population and are not clustered together. After all specimens of a species have been linked together (when they fall within limits) we identify population clusters hierarchically (c). Starting with the record with the most links (this can be thought of as the most central record) we cluster together that record and all linked records to form a population. This will represent the population in that species with the maximum sample size out of all subsequently identified populations. We chose to maximize sample size to ensure that the populations we identify could be applicable to genetic monitoring. After the first population is clustered, all records in that population are removed from contention and the process is repeated, to identify the second population, and continues until all records have been assigned a population. Afterwards, populations can be filtered based on the number of samples to identify which populations could be used to quantify different genetic EBVs.

**Figure S2** – Distribution and length of reconstructed series which could be used to monitor genetic EBVs through time. The temporal distribution of these series varies both by initial sampling date as well as by EBV (a), and while some series date as far back as 1831, most capture genetic change that has occurred post 1940 (b). Surprisingly, the average number of years between sampling events (visualized using thick colored lines in panel b) is 24 for genetic diversity, 22 for genetic differentiation, and 17 for inbreeding and effective population size, which is considerably longer than current best efforts to conduct genetic monitoring across many species (https://doi.org/10.1101/2025.08.01.663988 [preprint: not peer reviewed]). The number of species represented in series decreases sharply as the number of time points in a series increases (c). For genetic diversity for example, while 1571 species are represented in timeseries with minimum 3 reconstructed time points, only 74 species are represented by series with at least 6 time points.

**Section 6) SUPPLEMENTARY REFERENCES**

Anderson, J.T., Inouye, D.W., McKinney, A.M., Colautti, R.I., Mitchell-Olds, T., 2012. Phenotypic plasticity and adaptive evolution contribute to advancing flowering phenology in response to climate change. Proceedings of the Royal Society B: Biological Sciences 279, 3843–3852. https://doi.org/10.1098/rspb.2012.1051

Andrews, K.R., Good, J.M., Miller, M.R., Luikart, G., Hohenlohe, P.A., 2016. Harnessing the power of RADseq for ecological and evolutionary genomics. Nat Rev Genet 17, 81–92. https://doi.org/10.1038/nrg.2015.28

Arnaud-Haond, S., Alberto, F., Teixeira, S., Procaccini, G., Serrão, E.A., Duarte, C.M., 2005. Assessing Genetic Diversity in Clonal Organisms: Low Diversity or Low Resolution? Combining Power and Cost Efficiency in Selecting Markers. Journal of Heredity 96, 434–440. https://doi.org/10.1093/jhered/esi043

Bacles, C.F.E., Ennos, R.A., 2008. Paternity analysis of pollen-mediated gene flow for Fraxinus excelsior L. in a chronically fragmented landscape. Heredity 101, 368–380. https://doi.org/10.1038/hdy.2008.66

Bakker, F.T., Lei, D., Yu, J., Mohammadin, S., Wei, Z., van de Kerke, S., Gravendeel, B., Nieuwenhuis, M., Staats, M., Alquezar-Planas, D.E., Holmer, R., 2016. Herbarium genomics: plastome sequence assembly from a range of herbarium specimens using an Iterative Organelle Genome Assembly pipeline. Biological Journal of the Linnean Society 117, 33–43. https://doi.org/10.1111/bij.12642

Bradshaw, M.J., Carey, J., Liu, M., Bartholomew, H.P., Jurick, W.M., Hambleton, S., Hendricks, D., Schnittler, M., Scholler, M., 2023. Genetic time traveling: sequencing old herbarium specimens, including the oldest herbarium specimen sequenced from kingdom Fungi, reveals the population structure of an agriculturally significant rust. New Phytologist 237, 1463–1473. https://doi.org/10.1111/nph.18622

Brewer, G.E., Clarkson, J.J., Maurin, O., Zuntini, A.R., Barber, V., Bellot, S., Biggs, N., Cowan, R.S., Davies, N.M.J., Dodsworth, S., Edwards, S.L., Eiserhardt, W.L., Epitawalage, N., Frisby, S., Grall, A., Kersey, P.J., Pokorny, L., Leitch, I.J., Forest, F., Baker, W.J., 2019. Factors Affecting Targeted Sequencing of 353 Nuclear Genes From Herbarium Specimens Spanning the Diversity of Angiosperms. Front. Plant Sci. 10. https://doi.org/10.3389/fpls.2019.01102

Brown, M.J.M., Walker, B.E., Black, N., Govaerts, R., Ondo, I., Turner, R., Lughadha, E.N., 2023. rWCVP: A companion R package to the World Checklist of Vascular Plants. New Phytologist.

Brüniche–Olsen, A., Jones, M.E., Burridge, C.P., Murchison, E.P., Holland, B.R., Austin, J.J., 2018. Ancient DNA tracks the mainland extinction and island survival of the Tasmanian devil. Journal of Biogeography 45, 963–976. https://doi.org/10.1111/jbi.13214

Burbano, H.A., Gutaker, R.M., 2023. Ancient DNA genomics and the renaissance of herbaria. Science 382, 59–63. https://doi.org/10.1126/science.adi1180

Campos, P.E., Pruvost, O., Boyer, K., Chiroleu, F., Cao, T.T., Gaudeul, M., Baider, C., Utteridge, T.M.A., Becker, N., Rieux, A., Gagnevin, L., 2023. Herbarium specimen sequencing allows precise dating of Xanthomonas citri pv. citri diversification history. Nat Commun 14, 4306. https://doi.org/10.1038/s41467-023-39950-z

Chamberlain, S., Barve, V., Mcglinn, D., Oldoni, D., Desmet, P., Geffert, L., Ram, K., 2024. rgbif: Interface to the Global Biodiversity Information Facility API.

Chung, M.Y., Merilä, J., Li, J., Mao, K., López-Pujol, J., Tsumura, Y., Chung, M.G., 2023. Neutral and adaptive genetic diversity in plants: An overview. Front. Ecol. Evol. 11. https://doi.org/10.3389/fevo.2023.1116814

Compagnoni, A., Levin, S., Childs, D.Z., Harpole, S., Paniw, M., Römer, G., Burns, J.H., Che-Castaldo, J., Rüger, N., Kunstler, G., Bennett, J.M., Archer, C.R., Jones, O.R., Salguero-Gómez, R., Knight, T.M., 2021. Herbaceous perennial plants with short generation time have stronger responses to climate anomalies than those with longer generation time. Nat Commun 12, 1824. https://doi.org/10.1038/s41467-021-21977-9

Davis, C.C., 2023. The herbarium of the future. Trends in Ecology & Evolution 38, 412–423. https://doi.org/10.1016/j.tree.2022.11.015

Davis, C.C., Sessa, E., Paton, A., Antonelli, A., Teisher, J.K., 2024. Guidelines for the effective and ethical sampling of herbaria. Nat Ecol Evol. https://doi.org/10.1038/s41559-024-02544-z

Deck, J., Gaither, M.R., Ewing, R., Bird, C.E., Davies, N., Meyer, C., Riginos, C., Toonen, R.J., Crandall, E.D., 2017. The Genomic Observatories Metadatabase (GeOMe): A new repository for field and sampling event metadata associated with genetic samples. PLOS Biology 15, e2002925. https://doi.org/10.1371/journal.pbio.2002925

Dick, C.W., Hardy, O.J., Jones, F.A., Petit, R.J., 2008. Spatial Scales of Pollen and Seed-Mediated Gene Flow in Tropical Rain Forest Trees. Tropical Plant Biol. 1, 20–33. https://doi.org/10.1007/s12042-007-9006-6

Eckert, I., Bruneau, A., Metsger, D.A., Joly, S., Dickinson, T.A., Pollock, L.J., 2024. Herbarium collections remain essential in the age of community science. Nat Commun 15, 7586. https://doi.org/10.1038/s41467-024-51899-1

Evanno, G., Regnaut, S., Goudet, J., 2005. Detecting the number of clusters of individuals using the software structure : a simulation study. Molecular Ecology 14, 2611–2620. https://doi.org/10.1111/j.1365-294X.2005.02553.x

Franks, S.J., Sim, S., Weis, A.E., 2007. Rapid evolution of flowering time by an annual plant in response to a climate fluctuation. Proceedings of the National Academy of Sciences 104, 1278–1282. https://doi.org/10.1073/pnas.0608379104

Führding‐Potschkat, P., Kreft, H., Ickert‐Bond, S.M., 2022. Influence of different data cleaning solutions of point‐occurrence records on downstream macroecological diversity models. Ecology and Evolution 12, e9168. https://doi.org/10.1002/ece3.9168

GBIF.Org, 2024. Occurrence Download. https://doi.org/10.15468/DL.J9VX4F

Govaerts, R., Nic Lughadha, E., Black, N., Turner, R., Paton, A., 2021. The World Checklist of Vascular Plants, a continuously updated resource for exploring global plant diversity. Sci Data 8, 215. https://doi.org/10.1038/s41597-021-00997-6

Hijmans, R.J., Bivand, R., Forner, K., Ooms, J., Pebesma, E., Sumner, M.D., 2022. Package ‘terra.’ Maintainer: Vienna, Austria.

Hoban, S., Archer, F.I., Bertola, L.D., Bragg, J.G., Breed, M.F., Bruford, M.W., Coleman, M.A., Ekblom, R., Funk, W.C., Grueber, C.E., Hand, B.K., Jaffé, R., Jensen, E., Johnson, J.S., Kershaw, F., Liggins, L., MacDonald, A.J., Mergeay, J., Miller, J.M., Muller-Karger, F., O’Brien, D., Paz-Vinas, I., Potter, K.M., Razgour, O., Vernesi, C., Hunter, M.E., 2022. Global genetic diversity status and trends: towards a suite of Essential Biodiversity Variables (EBVs) for genetic composition. Biological Reviews 97, 1511–1538. https://doi.org/10.1111/brv.12852

Hodkinson, T.R., Waldren, S., Parnell, J.A.N., Kelleher, C.T., Salamin, K., Salamin, N., 2007. DNA banking for plant breeding, biotechnology and biodiversity evaluation. J Plant Res 120, 17–29. https://doi.org/10.1007/s10265-006-0059-7

Hughes, M., Möller, M., Edwards, T.J., Bellstedt, D.U., De Villiers, M., 2007. The impact of pollination syndrome and habitat on gene flow: a comparative study of two *Streptocarpus* (Gesneriaceae) species. American J of Botany 94, 1688–1695. https://doi.org/10.3732/ajb.94.10.1688

Kimura, M., 1962. ON THE PROBABILITY OF FIXATION OF MUTANT GENES IN A POPULATION. Genetics 47, 713–719. https://doi.org/10.1093/genetics/47.6.713

Kramer, A.T., Fant, J.B., Ashley, M.V., 2011. Influences of landscape and pollinators on population genetic structure: Examples from three Penstemon (Plantaginaceae) species in the Great Basin. American Journal of Botany 98, 109–121. https://doi.org/10.3732/ajb.1000229

Kremer, A., Ronce, O., Robledo‐Arnuncio, J.J., Guillaume, F., Bohrer, G., Nathan, R., Bridle, J.R., Gomulkiewicz, R., Klein, E.K., Ritland, K., Kuparinen, A., Gerber, S., Schueler, S., 2012. Long‐distance gene flow and adaptation of forest trees to rapid climate change. Ecology Letters 15, 378–392. https://doi.org/10.1111/j.1461-0248.2012.01746.x

Lang, P.L.M., Erberich, J.M., Lopez, L., Weiß, C.L., Amador, G., Fung, H.F., Latorre, S.M., Lasky, J.R., Burbano, H.A., Expósito-Alonso, M., Bergmann, D.C., 2024. Century-long timelines of herbarium genomes predict plant stomatal response to climate change. Nat Ecol Evol 8, 1641–1653. https://doi.org/10.1038/s41559-024-02481-x

Latorre, S.M., Lang, P.L.M., Burbano, H.A., Gutaker, R.M., 2020. Isolation, Library Preparation, and Bioinformatic Analysis of Historical and Ancient Plant DNA. Current Protocols in Plant Biology 5, e20121. https://doi.org/10.1002/cppb.20121

Lehtonen, J., Lanfear, R., 2014. Generation time, life history and the substitution rate of neutral mutations. Biol. Lett. 10, 20140801. https://doi.org/10.1098/rsbl.2014.0801

Lendemer, J., Thiers, B., Monfils, A.K., Zaspel, J., Ellwood, E.R., Bentley, A., LeVan, K., Bates, J., Jennings, D., Contreras, D., Lagomarsino, L., Mabee, P., Ford, L.S., Guralnick, R., Gropp, R.E., Revelez, M., Cobb, N., Seltmann, K., Aime, M.C., 2020. The Extended Specimen Network: A Strategy to Enhance US Biodiversity Collections, Promote Research and Education. BioScience 70, 23–30. https://doi.org/10.1093/biosci/biz140

Lososová, Z., Axmanová, I., Chytrý, M., Midolo, G., Abdulhak, S., Karger, D.N., Renaud, J., Van Es, J., Vittoz, P., Thuiller, W., 2023. Seed dispersal distance classes and dispersal modes for the European flora. Global Ecology and Biogeography 32, 1485–1494. https://doi.org/10.1111/geb.13712

Lou, R.N., Jacobs, A., Wilder, A.P., Therkildsen, N.O., 2021. A beginner’s guide to low‐coverage whole genome sequencing for population genomics. Molecular Ecology 30, 5966–5993. https://doi.org/10.1111/mec.16077

Lowe, A.J., Boshier, D., Ward, M., Bacles, C.F.E., Navarro, C., 2005. Genetic resource impacts of habitat loss and degradation; reconciling empirical evidence and predicted theory for neotropical trees. Heredity 95, 255–273. https://doi.org/10.1038/sj.hdy.6800725

Marinček, P., Wagner, N.D., Tomasello, S., 2022. Ancient DNA extraction methods for herbarium specimens: When is it worth the effort? Appl Plant Sci 10, e11477. https://doi.org/10.1002/aps3.11477

Millette, K.L., Fugère, V., Debyser, C., Greiner, A., Chain, F.J.J., Gonzalez, A., 2020. No consistent effects of humans on animal genetic diversity worldwide. Ecology Letters 23, 55–67. https://doi.org/10.1111/ele.13394

Neves, L.G., Davis, J.M., Barbazuk, W.B., Kirst, M., 2013. Whole-exome targeted sequencing of the uncharacterized pine genome. The Plant Journal 75, 146–156. https://doi.org/10.1111/tpj.12193

Padgham, M., Sumner, M.D., 2024. geodist: Fast, Dependency-Free Geodesic Distance Calculations.

Paz-Vinas, I., Jensen, E.L., Bertola, L.D., Breed, M.F., Hand, B.K., Hunter, M.E., Kershaw, F., Leigh, D.M., Luikart, G., Mergeay, J., Miller, J.M., Van Rees, C.B., Segelbacher, G., Hoban, S., 2021. Macrogenetic studies must not ignore limitations of genetic markers and scale. Ecology Letters 24, 1282–1284. https://doi.org/10.1111/ele.13732

Pearman, P.B., Broennimann, O., Aavik, T., Albayrak, T., Alves, P.C., Aravanopoulos, F.A., Bertola, L.D., Biedrzycka, A., Buzan, E., Cubric-Curik, V., Djan, M., Fedorca, A., Fuentes-Pardo, A.P., Fussi, B., Godoy, J.A., Gugerli, F., Hoban, S., Holderegger, R., Hvilsom, C., Iacolina, L., Kalamujic Stroil, B., Klinga, P., Konopiński, M.K., Kopatz, A., Laikre, L., Lopes-Fernandes, M., McMahon, B.J., Mergeay, J., Neophytou, C., Pálsson, S., Paz-Vinas, I., Posledovich, D., Primmer, C.R., Raeymaekers, J.A.M., Rinkevich, B., Rolečková, B., Ruņģis, D., Schuerz, L., Segelbacher, G., Kavčič Sonnenschein, K., Stefanovic, M., Thurfjell, H., Träger, S., Tsvetkov, I.N., Velickovic, N., Vergeer, P., Vernesi, C., Vilà, C., Westergren, M., Zachos, F.E., Guisan, A., Bruford, M., 2024. Monitoring of species’ genetic diversity in Europe varies greatly and overlooks potential climate change impacts. Nat Ecol Evol 8, 267–281. https://doi.org/10.1038/s41559-023-02260-0

Pearman, P.B., Broennimann, O., Albayrak, T., Alves, P.C., Bertola, L.D., Biedrzycka, A., Buzan, E., Cubric-Curik, V., Fedorca, A., Godoy, J.A., Hvilsom, C., Klinga, P., Konopiński, M.K., Kopatz, A., Laikre, L., Lopez Fernandez, M., Mergeay, J., Neophytou, C., Pálsson, S., Paz-Vinas, I., Posledovich, D., Rolečková, B., Ruņģis, D., Segelbacher, G., Kavčič Sonnenschein, K., Thurfjell, H., Träger, S., Vernesi, C., Vilà, C., Westergren, M., Zachos, F.E., Guisan, A., Bruford, M., 2023. Conserving genetic diversity during climate change: Niche marginality and discrepant monitoring capacity in Europe (preprint). Ecology. https://doi.org/10.1101/2023.03.24.533448

Poinar, H.N., Cooper, A., 2000. Ancient DNA: Do it right or not at all. Science 5482, 416.

Pollak, E., 1982. The rate of mutant substitution in populations with overlapping generations. Genet. Res. 40, 89–94. https://doi.org/10.1017/S0016672300018930

Savolainen, O., Pyhäjärvi, T., Knürr, T., 2007. Gene flow and local adaptation in trees. Annu. Rev. Ecol. Evol. Syst. 38, 595–619. https://doi.org/10.1146/annurev.ecolsys.38.091206.095646

Shaw, R.E., Farquharson, K.A., Bruford, M.W., Coates, D.J., Elliott, C.P., Mergeay, J., Ottewell, K.M., Segelbacher, G., Hoban, S., Hvilsom, C., Pérez-Espona, S., Ruņģis, D., Aravanopoulos, F., Bertola, L.D., Cotrim, H., Cox, K., Cubric-Curik, V., Ekblom, R., Godoy, J.A., Konopiński, M.K., Laikre, L., Russo, I.-R.M., Veličković, N., Vergeer, P., Vilà, C., Brajkovic, V., Field, D.L., Goodall-Copestake, W.P., Hailer, F., Hopley, T., Zachos, F.E., Alves, P.C., Biedrzycka, A., Binks, R.M., Buiteveld, J., Buzan, E., Byrne, M., Huntley, B., Iacolina, L., Keehnen, N.L.P., Klinga, P., Kopatz, A., Kurland, S., Leonard, J.A., Manfrin, C., Marchesini, A., Millar, M.A., Orozco-terWengel, P., Ottenburghs, J., Posledovich, D., Spencer, P.B., Tourvas, N., Unuk Nahberger, T., Van Hooft, P., Verbylaite, R., Vernesi, C., Grueber, C.E., 2025. Global meta-analysis shows action is needed to halt genetic diversity loss. Nature 638, 704–710. https://doi.org/10.1038/s41586-024-08458-x

Shi, X., 2004. INBREEDING AND INBREEDING DEPRESSION IN THE WILD LUPINE.

Sigwart, J.D., Schleuning, M., Brandt, A., Pfenninger, M., Saeedi, H., Borsch, T., Häffner, E., Lücking, R., Güntsch, A., Trischler, H., Töpfer, T., Wesche, K., Consortium, C., 2025. Collectomics – towards a new framework to integrate museum collections to address global challenges. NHCM 2, 1–20. https://doi.org/10.3897/nhcm.2.148855

Slarkin, M., 1985. Gene flow in natural populations. Annu. Rev. Ecol. Syst. 16, 393–430. https://doi.org/10.1146/annurev.es.16.110185.002141

Smith, S.A., Donoghue, M.J., 2008. Rates of Molecular Evolution Are Linked to Life History in Flowering Plants. Science 322, 86–89. https://doi.org/10.1126/science.1163197

Toczydlowski, R.H., Liggins, L., Gaither, M.R., Anderson, T.J., Barton, R.L., Berg, J.T., Beskid, S.G., Davis, B., Delgado, A., Farrell, E., Ghoojaei, M., Himmelsbach, N., Holmes, A.E., Queeno, S.R., Trinh, T., Weyand, C.A., Bradburd, G.S., Riginos, C., Toonen, R.J., Crandall, E.D., 2021. Poor data stewardship will hinder global genetic diversity surveillance. Proceedings of the National Academy of Sciences 118, e2107934118. https://doi.org/10.1073/pnas.2107934118

Viveiros-Moniz, M., García-Muñoz, A., Matias, L., Abdelaziz, M., Muñoz-Pajares, A.J., 2025. Monitoring biodiversity in the global change era: The importance of herbaria and genetic diversity. Perspectives in Plant Ecology, Evolution and Systematics 67, 125862. https://doi.org/10.1016/j.ppees.2025.125862

Waples, R.S., Hoelzel, A.R., Gaggiotti, O.E., Tiedemann, R., Palsbøll, P.J., Cipriano, F., Jackson, J.A., Bickham, J.W., Lang, A.R., 2018. Guidelines for genetic data analysis. Journal of Cetacean Research and Management 18, 33–80. https://doi.org/10.47536/jcrm.v18i1.421

Whiting, J.R., Booker, T.R., Rougeux, C., Lind, B.M., Singh, P., Lu, M., Huang, K., Whitlock, M.C., Aitken, S.N., Andrew, R.L., Borevitz, J.O., Bruhl, J.J., Collins, T.L., Fischer, M.C., Hodgins, K.A., Holliday, J.A., Ingvarsson, P.K., Janes, J.K., Khandaker, M., Koenig, D., Kreiner, J.M., Kremer, A., Lascoux, M., Leroy, T., Milesi, P., Murray, K.D., Pyhäjärvi, T., Rellstab, C., Rieseberg, L.H., Roux, F., Stinchcombe, J.R., Telford, I.R.H., Todesco, M., Tyrmi, J.S., Wang, B., Weigel, D., Willi, Y., Wright, S.I., Zhou, L., Yeaman, S., 2024. The genetic architecture of repeated local adaptation to climate in distantly related plants. Nat Ecol Evol 8, 1933–1947. https://doi.org/10.1038/s41559-024-02514-5

Willi, Y., Kristensen, T.N., Sgrò, C.M., Weeks, A.R., Ørsted, M., Hoffmann, A.A., 2022. Conservation genetics as a management tool: The five best-supported paradigms to assist the management of threatened species. Proceedings of the National Academy of Sciences 119, e2105076119. https://doi.org/10.1073/pnas.2105076119

Wright, S., 1931. Evolution in Mendelian populations. Genetics 16, 97–159. https://doi.org/10.1093/genetics/16.2.97

Yan, W., Wang, B., Chan, E., Mitchell-Olds, T., 2021. Genetic architecture and adaptation of flowering time among environments. New Phytologist 230, 1214–1227. https://doi.org/10.1111/nph.17229

Zizka, A., Antunes Carvalho, F., Calvente, A., Rocio Baez-Lizarazo, M., Cabral, A., Coelho, J.F.R., Colli-Silva, M., Fantinati, M.R., Fernandes, M.F., Ferreira-Araújo, T., Gondim Lambert Moreira, F., Santos, N.M.C., Santos, T.A.B., Dos Santos-Costa, R.C., Serrano, F.C., Alves Da Silva, A.P., De Souza Soares, A., Cavalcante De Souza, P.G., Calisto Tomaz, E., Vale, V.F., Vieira, T.L., Antonelli, A., 2020. No one-size-fits-all solution to clean GBIF. PeerJ 8, e9916. https://doi.org/10.7717/peerj.9916

Zizka, A., Silvestro, D., Andermann, T., Azevedo, J., Ritter, C.D., Edler, D., Farooq, H., Herdean, A., Ariza, M., Scharn, R., Svanteson, S., Wengstrom, N., Zizka, V., Antonelli, A., 2019. CoordinateCleaner: standardized cleaning of occurrence records from biological collection databases. Methods in Ecology and Evolution 7. https://doi.org/10.1111/2041-210X.13152
